# Fibrinogen in extracellular matrix remodeling: functional switching, source heterogeneity, and biomaterial translation

**DOI:** 10.3389/fcell.2026.1874458

**Published:** 2026-06-17

**Authors:** Chenxi Liao, Weiheng Lv, Jun Huang, Mariama Diallo, Zhihui Tian

**Affiliations:** 1 Department of Stomatology, Nanfang Hospital, Southern Medical University, Guangzhou, Guangdong, China; 2 School of Stomatology, Southern Medical University, Guangzhou, Guangdong, China

**Keywords:** bio-inspired materials, extracellular matrix, fibrinogen, functional switching, source heterogeneity, targeted therapy

## Abstract

Fibrinogen (FG), which is typically regarded as a circulating coagulation protein, is now supported by growing evidence as also serving as a context-dependent regulator of extracellular matrix (ECM) remodeling during tissue repair, inflammation, fibrosis, cancer progression, and biomaterial-mediated regeneration. In this review, we examine how FG activity is shaped by local concentration, polymerization state, proteolytic processing, post-translational modification, receptor availability, matrix mechanics, and cellular source. Rather than treating FG as a molecule that is uniformly reparative or pathogenic, we outline a functional-switching model wherein transient FG deposition and selected FG-derived fragments may support epithelial repair, endothelial barrier stabilization, regeneration, and immune resolution, whereas persistent FG/FN-rich matrices, excessive proteolysis, inflammatory receptor engagement, and altered mechanical cues are associated with chronic inflammation, fibrosis, vascular leakage, and tumor-supportive remodeling. We also consider source heterogeneity, through which systemic hepatocyte-derived FG is distinguished from locally produced or ectopically deposited FG in specialized tissue niches. Current evidence, which supports hepatocytes as the main source of circulating FG, also suggests that extrahepatic sources, including epithelial, endothelial, and tumor-associated cells, may influence local ECM behavior under disease-specific contexts. By contrast, the possibility of macrophage-derived FG remains unresolved, a situation that requires both transcript- and protein-level validation. Finally, we relate these biological principles to fragment-guided therapeutic design, receptor-selective blockade, source-aware targeting, and stimulus-responsive FG-based biomaterials, emphasizing that biomaterial translation should not simply incorporate FG as a passive scaffold but should instead program material stiffness, degradation kinetics, ligand exposure, and local fragment generation so that reparative signaling is favored while persistent inflammatory or fibrotic matrices are avoided. It is upon FG fragmentomics, spatial multi-omics, tunable biomaterial platforms, and lineage-resolved validation that future progress will depend, as these approaches will define when FG supports repair and when it contributes to pathological remodeling.

## Graphical Abstract

**Figure:**
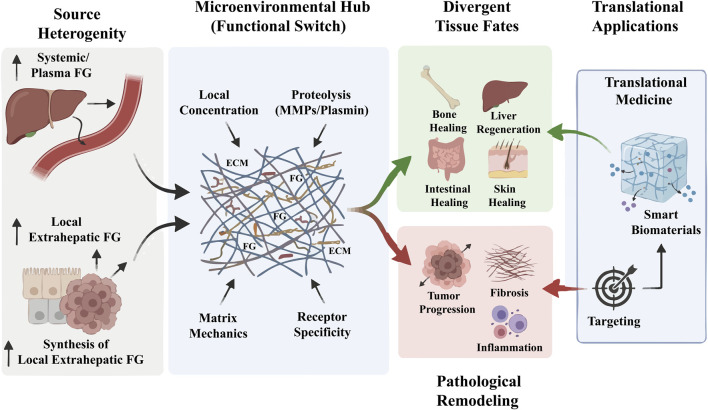

## 1 Introduction

The extracellular matrix (ECM) forms a three-dimensional scaffold around cells. It consists mainly of core matrisome proteins (∼40%, including collagens, proteoglycans, and abundant glycoproteins) and matrisome-associated proteins (∼60%, predominantly ECM regulators). This dynamic network is continuously remodeled and helps regulate cell survival, proliferation, and differentiation. Such remodeling is essential for cellular growth and development (Bonnans et al., 2014).

Although fibrinogen (FG) is synthesized mainly in the liver, smaller amounts are also expressed by extrahepatic tissues, including the brain, lungs, intestines, ovarian cells, trophoblast cells, and liver sinusoidal endothelial cells. Classically recognized as a plasma protein required for hemostasis, FG is increasingly being studied as an ECM glycoprotein involved in diverse biological processes.

Encoded by the FGA, FGB, and FGG genes, the respective mRNAs are translated into Aα, Bβ, and γ polypeptide chains. After post-translational modification, these chains assemble into a homodimeric (AαBβγ)_2_ structure stabilized by complex intra- and inter-chain disulfide bonds (Dobson et al., 2024). Structurally, the central E domain, which contains two copies each of fibrinopeptide A (FPA) and fibrinopeptide B (FPB), is flanked by two D domains, with extensions to the αC domain and the γ-chain C-terminus. FG contains multiple interaction motifs, some constitutively exposed and others unmasked during ECM remodeling, enabling binding to glycoproteins and soluble factors. As an ECM ligand, FG engages cell-surface receptors, especially integrins, and thereby regulates signaling pathways involved in adhesion, migration, proliferation, and survival. FG also acts as a substrate for ECM-degrading enzymes, including matrix metalloproteinases (MMPs) and plasmin, which can directly modulate matrix remodeling. Precise spatiotemporal regulation of these interactions supports tissue homeostasis, whereas dysregulated remodeling is associated with pathology; for example, aberrant FG deposition is a feature of tumor invasion and fibrotic disease.

Several knowledge gaps remain: (1) the molecular determinants of FG functional switching, including how local concentration, matrix mechanics, proteolysis, and receptor profiles shape reparative versus pathological outcomes; (2) the regulatory mechanisms and functional distinctness of extrahepatic, locally synthesized FG compared with plasma-derived FG; (3) the dynamic evolution of proteolytic FG fragments during disease progression; and (4) the interplay between FG and mechanotransduction pathways. To address these questions and support the development of targeted therapies, this review focuses on two analytical frameworks. First, we examine a microenvironment-driven functional-switching model in which FG output is shaped by local concentration, active fragment generation, and receptor availability. Second, we discuss the functional divergence between systemic (hepatic) and local (extrahepatic) FG synthesis. In some pathological niches, such as tumors or selected endothelial beds, ectopically synthesized FG may act independently of systemic coagulation and function as a localized paracrine matrix signal. Together, these frameworks map FG regulatory networks in matrix remodeling and may help guide the development of targeted interventions.

Throughout this review, we distinguish experimentally supported FG-ECM mechanisms from conceptual models proposed to guide future mechanistic and translational studies.

## 2 Mechanisms of fibrinogen-mediated extracellular matrix remodeling

ECM remodeling is a multifaceted process that includes matrix deposition (altering component abundance to shift biomechanical properties), biochemical modification (post-translational changes that alter structural features), proteolytic degradation (liberating bioactive fragments and matrix-bound factors), and force-mediated physical reorganization (aligning fibers to create migration tracks) (Winkler et al., 2020). The dynamic ECM state reflects the integration of these mechanisms. Within this matrix network, FG engages diverse ECM-associated proteins and cell-surface receptors to regulate cell adhesion and signal transduction. FG also serves as an important substrate for remodeling enzymes, including MMPs. Through these interactions, FG can influence the four remodeling mechanisms described above and thereby affect cellular behavior (Hastings et al., 2019).

### 2.1 Interactions with other ECM proteins

The core matrisome comprises collagens, proteoglycans, and glycoproteins. As a resident glycoprotein, FG interacts with these components and can directly or indirectly influence ECM remodeling. Its crosstalk with other non-collagenous glycoproteins is an important part of this structural reorganization.

#### 2.1.1 Glycoproteins

Non-collagenous glycoproteins are multidomain macromolecules that support structural integrity and direct ECM biological activity. During remodeling, reciprocal interactions of FG with fibronectin (FN) and laminin (LN) are among the best-characterized pathways.

##### 2.1.1.1 Fibronectin

Fibronectin (FN) acts as a central hub in ECM assembly because of its multidomain topology. A bidirectional regulatory relationship has been described between FG and FN: FG promotes FN matrix assembly, while FN reciprocally modulates FG deposition (Bonnans et al., 2014). This FG-FN co-assembly contributes to ECM remodeling by reorganizing matrix fibers, activating transmembrane signaling cascades, and shaping deposition patterns. By engaging cell-surface integrins, FG activates downstream kinases that induce cytoskeletal rearrangement and focal adhesion formation, creating a stable niche for cellular aggregation. In hepatocytes, for example, FG mediates physical adhesion and subsequent aggregation, which promotes the ordered assembly and cross-linking of surface FN (Li et al., 2022). Concurrently, the cell-anchored FG network facilitates FN membrane localization. This increases FN avidity for integrins and activates the FN-integrin αVβ1-Wnt/β-catenin axis involved in hepatic microenvironmental signaling (Li et al., 2022).

During vascular injury, FN acts as a molecular bridge that anchors FG to activated platelets and the damaged vessel wall, accelerating thrombus formation (Perutelli et al., 1992). This provisional matrix provides a structural template for further FN polymerization while reinforcing tissue barrier stability. Co-deposition of FG and FN also forms a provisional matrix at sites of niche remodeling. For instance, stressed tumor cells can induce local secretion and co-deposition of FG and FN, creating a protective niche that may favor survival and proliferation.

The functional heterogeneity of the FG-FN network, including the distinction between physiological healing and pathological remodeling, may be influenced by FN alternative splicing. Cellular fibronectin containing extra domain A (EDA-FN) is upregulated in chronic inflammation and the tumor microenvironment (TME) (Bonadio et al., 2024). Compared with plasma-derived wild-type FN, EDA-FN has been reported to show greater binding affinity for FG. Their co-assembly forms a denser, more stable fibrous network with increased resistance to MMP-mediated proteolysis (Bonadio et al., 2024). These altered physicochemical properties may favor persistent FG-FN matrix accumulation in tumors. By contrast, during physiological liver regeneration or acute wound healing, a wild-type FN-dominated provisional matrix is typically more dynamic and transient, allowing degradation and subsequent tissue remodeling.

In summary, FN splice isoforms may contribute to context-dependent FG-FN remodeling, but the EDA-FN-dependent switching model remains incompletely validated. Future studies using isoform-specific FN perturbation and high-resolution matrix imaging are needed to determine whether EDA-FN directly alters FG-FN co-assembly and downstream FG-integrin signaling.

##### 2.1.1.2 Laminin

Interactions between FG and laminin (LN) can modulate downstream signaling during ECM remodeling and influence cell adhesion, migration, and proliferation. In fibrin-based scaffolds, LN incorporation enhances interactions between human neural stem/progenitor cells (hNSPCs) and the matrix. Specifically, LN promotes hNSPC adhesion and differentiation on FG scaffolds, supporting its potential use in neural tissue engineering (Arulmoli et al., 2016). Similarly, FG and LN co-localize in intestinal epithelia, suggesting a cooperative role in epithelial adhesion and migration during wound healing (Seltana et al., 2022).

Beyond tissue repair, host-surface FG and LN can act as adhesins that facilitate pathogen binding. During Group B *Streptococcus* (GBS) colonization, for example, the fibrinogen-binding protein (Fbs) mediates host-cell adhesion, whereas the laminin-binding protein (Lmb) contributes to central nervous system tropism (Zeng et al., 2024; Shabayek and Spellerberg, 2018). These interactions help maintain bacteria-host contact and may facilitate the transition to invasive infection.

While FG-glycoprotein interactions are well documented, data regarding FG’s interplay with other ECM components, such as collagens and proteoglycans, remain limited. Emerging evidence suggests that FG may bridge cells to collagen fibers, providing an interfacial matrix that enables cell-mediated remodeling, such as collagen gel contraction (Reyhani et al., 2014). In addition, fibrin(ogen) can undergo FXIII- or tissue-transglutaminase-mediated cross-linking, stabilizing deposited fibrin(ogen) matrices and potentially increasing resistance to proteolysis (Poole et al., 2019; Poole et al., 2021).

### 2.2 Integrin-mediated cell-ECM interactions

During remodeling, cell-ECM interactions initiate actomyosin cytoskeleton reorganization through outside-in signaling and induce conformational changes in integrins. Subsequent inside-out signaling dynamically regulates integrin affinity for ECM ligands (Bonadio et al., 2024; Hantgan et al., 2010; Erb et al., 1997; Kopec et al., 2017; Yakovlev et al., 2022; Ugarova and Yakubenko, 2001; Bach et al., 1998). FG modulates ECM topology and signal transduction primarily through these integrin interactions. Specific integrin heterodimers shape FG output: αIIbβ3 mediates platelet aggregation and hemostasis; αMβ2 governs leukocyte adhesion, migration, and inflammatory amplification; and αVβ1 and α9β1 participate in cellular differentiation and polarity programs during liver regeneration and osteogenesis (Li et al., 2022; Kopec et al., 2017; Kidwai et al., 2016). This functional compartmentalization suggests that FG effects depend not on a single ligand interaction but on the combined influence of FG fragments, receptor subtypes, and cell lineages.

### 2.3 A proteolytic reprogramming switch and post-translational modifications

Rather than acting only as clearing enzymes for FG or fibrin, proteases such as matrix metalloproteinases (MMPs) and plasmin can function as remodeling switches. *In vivo* ECM degradation is driven mainly by three enzyme families: MMPs, plasmin, and cathepsins (Zhao et al., 2023). These enzymes maintain low baseline activity under physiological conditions but are upregulated during tissue healing, active remodeling, or inflammatory disease. Specific cleavage patterns influence not only FG clearance but also cryptic-site exposure, receptor availability, and the generation of fragments whose biological functions differ from those of the parent molecule (Yakovlev et al., 2022; Pechlivani et al., 2021; Nencini et al., 2024).

Post-translational modifications (PTMs), including oxidation, glycosylation, and citrullination, add another regulatory layer by altering FG structural stability, proteolytic susceptibility, and receptor interactions (Nencini et al., 2024; Risman et al., 2025; Nencini et al., 2025). For instance, oxidation reduces FG affinity for αIIbβ3 while increasing binding to αMβ2, shifting FG activity from hemostatic toward pro-inflammatory signaling (Nencini et al., 2025; Kaufmanova et al., 2021; Becatti et al., 2026). Glycosylation affects fibrin polymerization and plasmin sensitivity, whereas citrullination, which is increased in autoimmune diseases such as rheumatoid arthritis, modifies FG immunogenicity and clearance dynamics (Nencini et al., 2024; Tenopoulou, 2025).

The clinical relevance of FG citrullination is particularly evident in rheumatoid arthritis (RA), where citrullinated FG acts as both a modified coagulation substrate and an immunogenic matrix signal. PAD4, which can be expressed or released by neutrophils and monocytes under inflammatory conditions, can citrullinate extracellular FG in a calcium-dependent manner, generating neoepitopes that are selectively recognized by ACPAs, whereas native FG is generally not recognized (Gómez-Bañuelos et al., 2022; Sims et al., 2026; Bustos et al., 2025). This places citrullinated FG among the autoantigenic targets associated with the loss of tolerance to citrullinated proteins in RA (Bustos et al., 2025). ACPAs can promote inflammatory responses, suggesting that ACPA recognition of citrullinated FG may contribute to synovial inflammation, although the direct effects of ACPA-citrullinated FG immune complexes on synovial fibroblasts and macrophages remain insufficiently defined. Citrullination may also disturb coagulation-fibrinolysis balance: citrullinated SERPINs are enriched in RA, and citrullinated FG reduces fibrin clot lysis and alters clot mechanical properties, potentially promoting persistent fibrin-rich inflammatory matrices and abnormal ECM remodeling (Gómez-Bañuelos et al., 2022; Tilvawala et al., 2021). Clinically, ACPA responses to citrullinated antigens, including citrullinated FG, may contribute to diagnosis and patient stratification, particularly in ACPA-positive RA, while broader detection of citrullinated proteins may help complement current diagnostic strategies for seronegative or heterogeneous RA subsets (Bugatti et al., 2023). NETs and platelet-derived citrullinated antigens may provide additional sources of ACPA targets and support PAD inhibition as a potential therapeutic strategy (Sims et al., 2026; Xu et al., 2023). Thus, FG citrullination illustrates how a PTM can connect hemostatic imbalance, ECM remodeling, and autoimmunity, although its direct causal role in synovial matrix remodeling remains to be defined.

Nevertheless, the dynamic evolution and regulatory impact of these PTMs in disease contexts remain incompletely characterized and require further systematic study using mass spectrometry-based proteomics (Nencini et al., 2024; Risman et al., 2025; Tenopoulou, 2025).

#### 2.3.1 Matrix metalloproteinases (MMPs)

Soluble and membrane-anchored matrix metalloproteinases (MMPs) cleave ECM components with broad substrate specificity. After activation by proteolytic cleavage or thiol oxidation, they degrade diverse structural proteins. Several MMPs, including MMP-1, MMP-2, MMP-3, MMP-7, and MMP-9, have been reported to cleave or structurally modify fibrinogen or fibrin under defined experimental conditions (Monaco et al., 2007; Kumar et al., 2020; Hiller et al., 2000; Bini et al., 1999). MMP-1 and MMP-2 provide prominent examples (Pechlivani et al., 2021). Biochemical studies further show that MMP-3 and MMP-7 can degrade fibrinogen, supporting a direct role for selected MMPs in fibrinogen remodeling (Bini et al., 1999). MMP-9 can bind fibrin and participate in fibrin proteolysis under selected experimental conditions (Kumar et al., 2020; Makowski and Ramsby, 1999). Under selected pathological or remodeling conditions, MMPs may contribute to fibrin clearance by directly degrading fibrin(ogen) or by cooperating with plasmin-linked proteolytic cascades (Makowski and Ramsby, 1999; Ramos-DeSimone et al., 1999; Bini et al., 1996).

During tissue healing, limited MMP-mediated proteolysis removes excess provisional matrix and releases repair signals. For instance, after acetaminophen-induced liver injury, FG promotes regeneration by upregulating MMP-12 in a leukocyte αMβ2-dependent manner (Kopec et al., 2017). During chronic inflammation, fibrosis, or tumor progression, however, dysregulated MMP activity contributes to aberrant remodeling and generates bioactive FG fragments with pro-inflammatory or pro-angiogenic properties. Fragment E, for example, stimulates endothelial cell proliferation and migration and thereby supports angiogenesis (Bootle-Wilbraham et al., 2001). Conversely, other cleavage events yield anti-angiogenic fragments such as alphastatin, indicating that the FG proteolytic fragment repertoire contains complex biological information (Staton et al., 2004).

#### 2.3.2 Plasmin

The serine protease plasmin is a central effector of fibrinolysis and degrades both fibrinogen and insoluble fibrin (Chapin and Hajjar, 2015). By removing fibrin-rich deposits, plasmin contributes to provisional matrix clearance during tissue remodeling (Chapin and Hajjar, 2015). Within the ECM, FG adsorption onto specific surfaces facilitates local plasminogen activation. FG degradation products can also indirectly modulate the release of growth factors and their receptors, thereby influencing ECM remodeling (Nencini et al., 2024). Active plasmin triggers a proteolytic cascade by activating downstream MMPs, accelerating ECM degradation and liberating sequestered growth factors (GFs) involved in cellular proliferation, migration, and differentiation (Minciacchi et al., 2024). Plasmin also cleaves protease-activated receptors (PARs), initiating intracellular signaling cascades that further direct cellular behavior (Medcalf and Keragala, 2021).

Plasmin-generated FG fragments can exhibit biological activities that differ markedly from those of the parent molecule. The Bβ15-42 peptide and its derivative FX06 engage VE-cadherin-associated pathways to stabilize endothelial junctions and reduce vascular leakage (Bach et al., 1998; Gröger et al., 2009). Other specific fragments show pro-endothelial migratory or pro-inflammatory properties (Yakovlev et al., 2022; Bootle-Wilbraham et al., 2001). Thus, proteolysis is not simply the endpoint of FG clearance; it can convert a matrix scaffold into a reservoir of active signaling peptides. In this context, we use “FG fragmentomics” to describe systematic profiling of FG-derived proteolytic fragments, including their cleavage origins, PTM states, receptor-binding profiles, spatial distribution, and biological functions across physiological and pathological remodeling stages. This approach may help distinguish protective fragments, such as Bβ15-42/FX06-like peptides, from pro-inflammatory or pro-angiogenic fragments, such as Fragment E, and may support disease-stage-specific biomarker discovery and therapeutic design.

To delineate this structural-to-signaling transition, Table 1 summarizes major proteolytic FG fragments, their cleaving enzymes, receptor targets, and microenvironmental remodeling effects across physiological and pathological contexts.

**TABLE 1:** Functional differentiation and microenvironmental remodeling effects of major fibrinogen proteolytic fragments.

Active proteolytic fragment	Primary cleaving Enzyme(s)	Receptors/Targets	Microenvironmental biological effects	Predominant pathological/Physiological contexts	Evidence level/Translational caution
Bβ15-42 (and its mimetic peptide FX06)	Thrombin cleavage of FPB unmasks the β15-42 domain; plasmin further releases N-terminal fragments like Bβ1-42. FX06 is a functional mimetic peptide (Hurlet-Jensen et al., 1983; Riedel et al., 2011; Jennewein et al., 2011)	Classically targets VE-cadherin; additionally modulates β15-42-dependent recognition/signaling at the platelet-fibrin interface (Bach et al., 1998; Gröger et al., 2009; Petzelbauer et al., 2005; Chen et al., 1988; Hamaguchi et al., 1993)	Stabilizes the endothelial barrier, attenuates leukocyte transendothelial migration and vascular leakage, and mitigates ischemia-reperfusion injury; modulates platelet spreading on fibrin (Gröger et al., 2009; Petzelbauer et al., 2005; Chen et al., 1988; Hamaguchi et al., 1993)	Shock/sepsis-induced capillary leakage, cardio-renal ischemia-reperfusion, transplantation, and thrombo-inflammatory conditions driven by endothelial injury (Gröger et al., 2009; Jennewein et al., 2011; Petzelbauer et al., 2005)	Mechanistically and clinically studied; FX06 clinical efficacy remains uncertain because the F.I.R.E. trial did not meet its primary endpoint
Fragment E	A terminal product of progressive plasmin-mediated fibrin(ogen) cleavage (X→Y + D; Y→E + D) (Jennewein et al., 2011; Budzynski and Marder, 1977)	Acts on activated endothelial cells, endothelial progenitor cells, macrophages, and fibroblasts; acts synergistically with integrin adhesion axes and TGF-β signaling (Bootle-Wilbraham et al., 2001; Lee et al., 1999; Fuchs et al., 2020)	Promotes endothelial proliferation, migration, and differentiation; induces pro-inflammatory mediators (e.g., IL-6); amplifies TGF-β-driven myofibroblast activation and recruitment (Bootle-Wilbraham et al., 2001; Lee et al., 1999; Fuchs et al., 2020)	Wound healing, granulation tissue formation, angiogenesis, post-inflammatory fibrosis, and tumor-associated microenvironmental remodeling (Bootle-Wilbraham et al., 2001; Jennewein et al., 2011; Lee et al., 1999; Fuchs et al., 2020)	Experimentally supported, but effects are context-dependent and require disease-stage-specific validation
Fibrinopeptides A/B (FPA/FPB)	Primarily released via thrombin cleavage; FPB is released relatively early during surface-bound fibrin(ogen) assembly (Riedel et al., 2011; Jennewein et al., 2011)	FPB/Bβ1-42 exhibits chemotactic activity for neutrophils and fibroblasts; FPA directly activates endothelial inflammatory signaling (high-affinity receptors remain incompletely characterized) (Jennewein et al., 2011; Kay et al., 1974)	Serves as a coagulation activation marker; promotes inflammatory cell recruitment and early reparative cell migration; FPA induces endothelial CRP/inflammatory pathways (Riedel et al., 2011; Jennewein et al., 2011; Kay et al., 1974)	Acute thrombosis, the early inflammation-repair phase post-injury, and vascular inflammatory contexts such as atherosclerosis (Riedel et al., 2011; Kay et al., 1974)	Established coagulation cleavage products; direct ECM-remodeling receptor mechanisms remain incompletely defined
Alphastatin (24-amino acid fragment of the α-chain)	Derived from the N-terminal 24 aa of the Aα chain; current activity evidence relies on synthetic peptides, while its native *in vivo* generation pathway remains undetermined (Staton et al., 2004; Jennewein et al., 2011)	Primarily targets activated endothelial cells and their VEGF/bFGF response axes; direct molecular receptors remain unelucidated (Staton et al., 2004)	Inhibits endothelial migration, tube formation, and tumor-associated neoangiogenesis, with anti-angiogenic effects (Staton et al., 2004)	Tumor angiogenesis, inflammatory neovascularization, and conditions in which suppression of aberrant vascular responses may be beneficial (Staton et al., 2004)	Mainly synthetic peptide-based evidence; native in vivo generation and direct receptors remain unresolved
Degradation Intermediates Harboring Cryptic Epitopes	Thrombin-induced conformational shifts, further cleavage by plasmin and inflammatory proteases, or local matrix assembly unmasks previously shielded cryptic epitopes (Jennewein et al., 2011; Lawrence and Simpson-Haidaris, 2004; Flick et al., 2004; Adams et al., 2007; Ryu et al., 2018; Pereira et al., 2002)	Representative targets include VE-cadherin, leukocyte αMβ2 (Mac-1), and selected FDP-associated inflammatory receptor pathways (Bach et al., 1998; Jennewein et al., 2011; Lawrence and Simpson-Haidaris, 2004; Flick et al., 2004; Adams et al., 2007; Ryu et al., 2018)	Triggers leukocyte adhesion/activation, permeability alterations, cytoskeletal rearrangement, and neuro/articular inflammatory amplification; blocking specific cryptic epitopes attenuates inflammation while preserving hemostasis (Lawrence and Simpson-Haidaris, 2004; Flick et al., 2004; Adams et al., 2007; Ryu et al., 2018)	Rheumatoid arthritis, multiple sclerosis/neurodegenerative diseases, tumor stroma, and injured epithelial-mesenchymal interfaces characterized by “coagulation-inflammation” niches (Lawrence and Simpson-Haidaris, 2004; Flick et al., 2004; Adams et al., 2007; Ryu et al., 2018; Simpson-Haidaris and Rybarczyk, 2001; Wu et al., 2024)	Strong mechanistic rationale, but heterogeneous species require epitope-, disease-, and stage-specific validation

Abbreviations: FPA, fibrinopeptide A; FPB, fibrinopeptide B; VEGF, vascular endothelial growth factor; bFGF, basic fibroblast growth factor; CRP, C-reactive protein; FDP, fibrin/fibrinogen degradation products; MMPs, matrix metalloproteinases. The dynamic generation and biological output of these fragments depend strongly on the local proteolytic network, including thrombin, plasmin, and MMPs, and may shape the transition from physiological tissue repair to pathological inflammation.

### 2.4 Matrix mechanics and cellular origins in FG-integrin signaling

#### 2.4.1 The mechanosensing framework: stiffness, FN co-assembly, and cellular origins

The following section presents a hypothesis-generating mechanosensing framework rather than a fully validated FG-specific pathway. Matrix stiffness, FN assembly, integrin tension, and YAP signaling are established components of ECM mechanotransduction; however, direct evidence linking these processes into a unified FG-FN-integrin switching axis remains limited. Therefore, the FG-specific connections proposed below should be interpreted as a conceptual framework to guide future experiments.

Matrix stiffness regulates integrin clustering and downstream signaling intensity via mechanotransduction. In low-stiffness matrices, integrin signaling is often associated with migratory programs, whereas high-stiffness environments can favor cell proliferation and ECM synthesis (Ting et al., 2021; Dalton and Lemmon, 2021). Given FG’s reliance on integrin networks, these stiffness-dependent mechanisms may provide a microenvironmental context for FG functional switching across pathological stages, although direct FG-specific stiffness thresholds remain insufficiently defined.

Fibronectin (FN), a key mechanosensitive ECM effector (Dalton and Lemmon, 2021), undergoes fibrillar assembly in a stiffness-dependent manner. In stiff microenvironments, fibroblasts assemble denser and more highly aligned FN fibrillar networks enriched with the EDA domain (Patten et al., 2025). Mechanical tension can also induce conformational changes and PTMs in FN, thereby altering its biomechanical properties and integrin-binding affinity (Li W. et al., 2024). Because FG and FN frequently co-deposit during tissue remodeling, FN mechanoresponsiveness may indirectly influence FG-integrin signaling by modulating FG-FN co-assembly architecture, ligand spacing, and matrix accessibility. In parallel, YAP, as a central mechanotransduction hub (Patten et al., 2025), may participate in the cellular interpretation of FG-FN matrix mechanics; however, whether YAP directly couples FG-FN co-assembly to specific FG-integrin outputs remains to be experimentally established. Together, these findings support a testable model in which matrix stiffness may bias FG-integrin activation by remodeling ECM architecture, particularly through FN assembly.

Regarding FG cellular origins, spatial multi-omics and single-cell RNA sequencing (scRNA-seq) provide high-resolution tools to distinguish systemic FG deposition from local FG synthesis. In fibrotic niches, specific macrophage subsets have been reported to upregulate FN1 expression (Rudnik et al., 2021) and contribute to fibrosis progression (Aran et al., 2019). However, these studies support a role for macrophage subsets in FN-rich fibrotic niche remodeling rather than directly demonstrating macrophage synthesis of FG. Thus, whether defined profibrotic macrophage subsets contribute to local FG production remains unresolved and requires direct transcript- and protein-level validation.

We therefore propose a working model in which matrix stiffness may influence FG-integrin coupling through FG-FN co-deposition, potentially biasing cells toward migratory or proliferative/synthetic phenotypes. Integrating stiffness-tunable hydrogels with scRNA-seq, spatial transcriptomics, and spatial proteomics will help test this model and define whether FG-specific mechanotransduction pathways can be therapeutically targeted.

#### 2.4.2 Synthetic biomaterial platforms for interrogating FG-ECM mechanodynamics

To test the mechanosensing framework proposed in Section 2.4.1, engineered synthetic biomaterials provide useful tools for decoupling complex biophysical variables.

1. Single-Molecule Tension Quantification and Stiffness-Tunable Platforms: Hydrogels with controlled elastic moduli, such as polyacrylamide (PA) or polyethylene glycol (PEG) systems, enable mechanical cues to be isolated from biochemical signaling. Molecular tension probes that modulate mechanical tolerance at the cell-matrix interface allow direct manipulation of bonding forces between integrins and fibronectin (FN) (Han et al., 2024). Reductions in these forces have been shown to inhibit cell spreading and focal adhesion kinase (FAK) phosphorylation (Han et al., 2024). Atomic force microscopy (AFM) and single-molecule force spectroscopy (SMFS) also permit quantification of piconewton (pN)-scale force thresholds for specific integrin subtypes (e.g., α4β1, αvβ1, and α5β1) binding to FG or FN (Chen et al., 2024; Jo et al., 2022). These techniques provide a mechanistic basis for studying how substrate stiffness and molecular tension regulate exposure of cryptic domains.
2. Dynamic and 3D Platforms for Tracking Mechanical Memory: Dynamic, reversibly tunable hydrogels combined with tension-sensing technologies can recapitulate the progressive stiffening characteristic of fibrotic diseases. Studies indicate that integrin-mediated adhesion alters the perception of ECM stiffness in human mesenchymal stem cells (hMSCs) through a “mechanical memory” effect driven by hysteresis in F-actin kinetics (Zhang et al., 2023). Integrating FRET-based tension sensors into 3D biomaterials also enables real-time visualization of integrin tension at single-molecule resolution. These studies indicate that integrin tension magnitudes scale inversely with substrate stiffness, providing a quantitative biophysical parameter for evaluating cellular responses to an evolving matrix environment (Sarkar et al., 2023).
3. Dual-Functionalized Matrices for Dissecting Integrin Competition: Synthetic interfaces with controlled ligand ratios allow competitive and cooperative interactions during FG-FN co-assembly to be examined. At low FN concentrations, αvβ3 integrins and FG primarily promote signaling; however, increased FN density recruits α5β1 integrins to govern cell migration and matrix remodeling (Feng et al., 2025; Miyamoto et al., 2022). Experiments using nanopatterned hydrogels with α5β1-specific peptidomimetics show that collective keratinocyte migration is regulated by α5β1 density, independent of substrate rigidity (Di Russo et al., 2021). In 3D hepatic spheroid models, the presence of FG and FN enhances cell aggregation and facilitates FN surface assembly, subsequently activating the Wnt/β-catenin pathway (Li et al., 2022), while FN blockade impairs collagen fibrillogenesis (Ma et al., 2026). These platforms can model how pathological shifts in ECM composition, such as EDA-FN upregulation, redirect intracellular signaling programs (Peng et al., 2022).

Table 2 summarizes representative biomaterial and mechanobiology platforms that can be used to interrogate FG-ECM mechanodynamics, including tunable fibrin/fibrinogen matrices, molecular tension probes, single-molecule force spectroscopy, dynamic mechanosensing substrates, tension sensors, nanopatterned ligand-presentation hydrogels, and FG-FN co-assembly 3D models.

**TABLE 2:** Synthetic biomaterial platforms for interrogating FG-ECM mechanodynamics.

Platform type	Representative implementation	Main tunable/Measurable parameter(s)	Research findings	Relevance to FG-ECM mechanodynamics
Stiffness-tunable fibrin/fibrinogen matrices	3D fibrin matrices formed with different fibrinogen/thrombin compositions	Matrix stiffness; fibril architecture; cell-mediated stiffening	Increasing fibrinogen concentration increases 3D fibrin matrix stiffness; embedded fibroblasts further remodel matrix mechanics (Duong et al., 2009)	Provides a direct FG/fibrin platform to test how bulk stiffness alters cell behavior in an FG-rich matrix
Single-molecule tension probe platforms	Molecular DNA-based tension probes coupled to ECM ligands	Integrin-ligand rupture force; cell spreading; focal adhesion formation; FAK phosphorylation	Lower integrin-ligand force tolerance suppresses spreading, focal adhesions, and FAK signaling (Han et al., 2024)	Suitable for testing how FG- versus FN-mediated adhesion transmits force at the cell-matrix interface
AFM/single-molecule force spectroscopy	Subtype-specific beta1 integrin mechanics measured at single-molecule scale	Force threshold for integrin activation and spreading; subtype-specific mechanics	Integrin alpha4beta1 and RGD-binding integrins (alphaVbeta1 and alpha5beta1) show distinct force thresholds and cytoskeletal outputs (Jo et al., 2022)	Useful for comparing the force requirements of FG-binding versus FN-binding integrin programs
Dynamic/reversible mechanosensing substrates	DNA-driven reversible RGD presentation on substrates of defined stiffness	Cyclic adhesion-detachment; actin hysteresis; nuclear mechanosensing; stiffness memory	Cyclic integrin adhesion changes how hMSCs interpret ECM stiffness through transient mechanical memory (Zhang et al., 2023)	Can be adapted to test whether dynamic FG exposure/deposition shifts cells from migratory to matrix-synthetic states
FRET-based integrin tension sensors	Molecular or ratiometric integrin tension sensors in living cells	Integrin tension magnitude; force distribution within focal adhesions	Integrin forces can be visualized with single-molecule sensitivity; newer ratiometric sensors better isolate tension magnitude (Sarkar et al., 2023; Morimatsu et al., 2013)	Provides a quantitative readout for FG-integrin and FG-FN-integrin force transmission
Nanopatterned ligand-presentation hydrogels	alpha5beta1-specific peptidomimetic nanopatterns on hydrogels	Ligand nanospacing; collective migration; independence from substrate rigidity	Keratinocyte collective migration depends on optimal alpha5beta1 nanospacing and can become largely independent of bulk rigidity (Di Russo et al., 2021)	Supports the idea that receptor organization, not only bulk stiffness, may control FG/FN functional switching
FG-FN co-assembly organotypic 3D models	Hepatic spheroids supplemented with fibrinogen and fibronectin	Cell aggregation; FN assembly; polarity; beta-catenin signaling; hepatic function	Fibrinogen promotes cell aggregation and FN assembly on the cell surface, improving hepatic spheroid function (Li et al., 2022)	Directly supports the use of 3D multicellular models to dissect FG-FN co-assembly in regenerative niches

AFM, atomic force microscopy; ECM, extracellular matrix; FAK, focal adhesion kinase; FG, fibrinogen; FN, fibronectin; FRET, fluorescence resonance energy transfer; hMSCs, human mesenchymal stem cells; RGD, Arg-Gly-Asp.

### 2.5 Core regulatory nodes of FG functional switching

**FIGURE 1:**
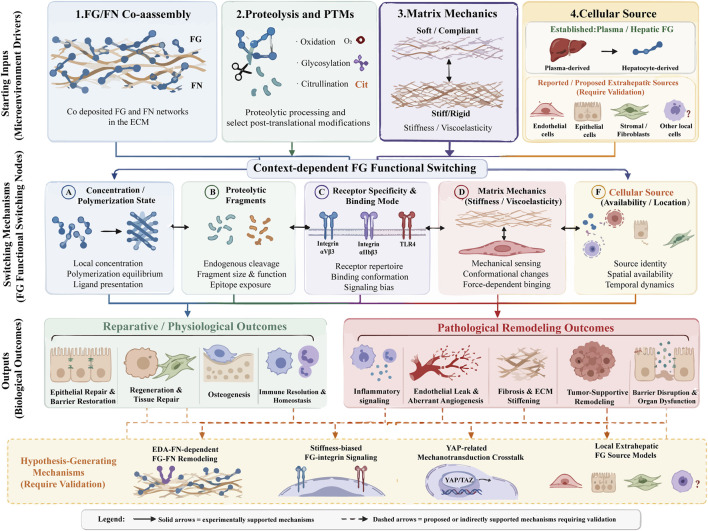
Context-dependent functional switching of fibrinogen during ECM remodeling. FG output is shaped by local concentration, proteolysis, matrix mechanics, receptor availability, and cellular source. Controlled FG deposition and protective fragments support repair, whereas persistent FG/FN-rich matrices and inflammatory receptor engagement may promote pathological remodeling. Solid arrows indicate experimentally supported links; dashed arrows indicate proposed or indirectly supported mechanisms requiring further validation.

The preceding sections discussed FG interactions with ECM proteins (Section 2.1), integrin receptors (Section 2.2), proteolytic regulators (Section 2.3), and matrix mechanics (Section 2.4). Rather than operating in isolation, these mechanisms can be organized into a context-dependent regulatory network that shapes FG functional switching across ECM niches (Figure 1). This network includes four interacting nodes whose relative contributions may vary by tissue context, disease stage, proteolytic environment, and receptor repertoire.

1. Concentration and Polymerization: Local FG density and physical state influence signaling pathway selection, cell migration, and barrier stability (detailed in Section 3.2.2).
2. Proteolysis: Proteolytic cleavage converts FG into a repertoire of functionally distinct fragments, mediating the transition from a structural scaffold to a signaling reservoir (detailed in Section 2.3).
3. Matrix Mechanics: Matrix stiffness and viscoelasticity may influence FG-integrin interactions indirectly through mechanotransduction, FN co-assembly, and cytoskeletal tension, thereby biasing cellular responses (detailed in Section 2.4).
4. Receptor Specificity: The combinatorial interplay among receptor availability, FG fragments, matrix context, and cell lineage shapes FG’s biological output.

For instance, reparative contexts such as liver regeneration are associated with controlled proteolysis, protective fragments such as Bβ15-42, αVβ1/α9β1-related signaling, and a degradable provisional matrix. By contrast, pathological contexts such as fibrosis may involve excessive MMP activation, Fragment E accumulation, sustained inflammatory receptor activation, and a rigid or poorly degradable matrix. In parallel, FG source heterogeneity, including plasma-derived hepatic FG versus locally synthesized extrahepatic FG from reported sources such as LSECs, intestinal epithelia, or tumor cells, may influence these regulatory nodes through spatial distribution, local concentration, and tissue-specific modifications. However, proposed sources without direct transcript- and protein-level validation should be treated as unresolved questions rather than established mechanisms. To date, LSEC-derived FG in liver fibrosis remains one of the better-characterized examples of local FG production and is discussed further in Section 4.3.

Figure 1 schematically summarizes these regulatory nodes and distinguishes experimentally supported mechanisms from proposed or indirectly supported links.

## 3 Function of fibrinogen in tissue healing and regeneration

FG can promote healing in diverse tissues, including bone, liver, intestine, and skin, by modulating ECM remodeling, which provides structural support and directs cellular behaviors such as motility and growth (Bonnans et al., 2014). As an ECM constituent, local FG synthesis and reorganization can activate signaling pathways involved in tissue regeneration.

### 3.1 Osteogenesis: osteogenic signaling and osteoimmunological remodeling

As a microenvironmental matrix protein, FG has regulatory potential in bone regeneration. In human embryonic stem cell (hESC) models, FG promotes osteogenesis by engaging integrin α9β1, which phosphorylates and activates the SMAD1/5/8 pathway. This cascade upregulates the osteogenic transcription factor Runx2 and induces ECM mineralization in osteoblasts (Kidwai et al., 2016). This FG-mediated osteogenic network appears to include redundancy, because FG retains osteoinductive capacity even when the α9β1-SMAD axis is blocked, suggesting compensatory receptors or alternative mechanotransduction pathways. Beyond direct stem-cell induction, FG’s physical conformation and immunomodulatory effects may also contribute to osteogenesis. For instance, FG deposited in a globular conformation on titanium alloy scaffolds promotes osteogenic differentiation and mineralization through cell-autonomous endogenous BMP signaling (Horasawa et al., 2015).

FG also coordinates osteoimmunological networks. On FG-supplemented chitosan membranes, polarized macrophages secrete osteogenic and angiogenic factors such as PDGF-BB, BMP-5, BMP-7, and FGF-7, with stronger tissue-inductive effects than bare chitosan (Maciel et al., 2014). *In vivo*, distinct spatial assemblies of FG scaffolds mediate differential immune recruitment: two-dimensional adsorbed FG (Fg-Ads) chemoattracts B lymphocytes and macrophages while upregulating systemic TGF-β1, whereas three-dimensional FG (Fg-3D) induces local pro-inflammatory cytokine (IL-6, IL-8) release (Vasconcelos et al., 2016). These findings suggest that FG extends beyond simple adhesion and can amplify endogenous osteogenic signals through receptor engagement and immune modulation. We therefore propose FG as a potential “BMP-sparing platform”: pre-programming integrin recognition and macrophage responses through FG coatings may reduce the required dose of exogenous rhBMP-2, potentially mitigating risks and costs associated with supraphysiological BMP concentrations (Horasawa et al., 2015; Maciel et al., 2014; Vasconcelos et al., 2016; Santos et al., 2013).

### 3.2 Organ regeneration

#### 3.2.1 Liver: FG-FAK signaling and cell aggregation

In acetaminophen-induced liver injury models, deposited fibrin(ogen) acts as an important niche signal. By binding leukocyte integrin αMβ2, it upregulates MMP-12 expression and contributes to inflammation resolution and tissue repair (Kopec et al., 2017). *In vitro* HepaRG models clarify this regenerative receptor cascade: FG engages hepatocyte integrin αVβ1, phosphorylates focal adhesion kinase (FAK), and activates the β-catenin pathway through GSK3β modulation. Physically, FG self-assembles into a mesh-like scaffold that promotes hepatocyte aggregation, strengthens intercellular connections, and synergizes with FN to guide hepatocyte polarity (Li et al., 2022). In murine partial hepatectomy (PHx) models, FG contributes to systemic regenerative networks by recruiting platelets and regulating tissue factor (TF)-driven signaling in the remnant liver, thereby limiting potentially excessive regenerative stimuli (Groeneveld et al., 2019). These findings support dual niche functions for FG during regeneration: scaffold formation and biochemical signaling.

#### 3.2.2 Intestine: dual repair effects and source considerations

In the intestinal microenvironment, FG has context-dependent repair-injury effects on the mucosal barrier. As a component of the intestinal epithelial basement membrane, FG accelerates epithelial monolayer healing through the PI3K pathway (Seltana et al., 2022). Although intestinal epithelia synthesize FG locally, functional distinctions between local and plasma-derived FG during repair likely depend more on spatial distribution than on distinct molecular mechanisms (Seltana et al., 2022). FG’s biological effects are especially sensitive to deposition density and polymerization state. Low-density or soluble FG preferentially activates the PI3K axis for epithelial repair. By contrast, pathological high-density polymerization, as may occur in inflammatory bowel disease, can induce receptor over-clustering, leading to barrier dysfunction and inflammatory amplification (Zhang et al., 2021). In radiation-induced intestinal injury, FG deficiency (Fib−/−) alleviates structural damage and inflammatory infiltration, supporting a pathogenic role for excessive FG deposition (Wang et al., 2017). This context dependency extends to other organs; for instance, in obese ApoE-deficient models, FG protects lung function by inhibiting MMP-9 activation and Syndecan-1 cleavage, suggesting that tissue-specific niches shape FG output.

### 3.3 Skin wound healing: a three-phase immunomodulatory cascade

FG-regulated skin wound healing involves a spatiotemporally ordered cascade comprising hemostasis/inflammation, tissue proliferation, and matrix remodeling/maturation. After tissue injury, thrombin rapidly cleaves extravasated plasma FG to polymerize fibrin and form the initial hemostatic clot (Weisel and Litvinov, 2013). Beyond serving as a physical hemostatic barrier, this provisional fibrin matrix functions as a bioactive scaffold that engages endothelial cells, fibroblasts, keratinocytes, and infiltrating leukocytes. Through receptor-mediated transmembrane signaling, this network initiates repair programs, including directed cell migration, proliferation, and angiogenesis (Kearney et al., 2022). Neutrophils and monocytes also undergo transendothelial migration (Medrano-Bosch et al., 2023); in this setting, fibrin(ogen) modulates monocyte and macrophage activity and thereby influences the transition from inflammation to tissue repair (Hsieh et al., 2017).

During the subsequent proliferation phase, FG contributes to angiogenesis and matrix reconstruction. The FG αC domain induces endothelial integrin clustering, which activates focal adhesion kinase (FAK) and extracellular signal-regulated kinase 1/2 (ERK1/2) pathways. This axis enhances endothelial migration and proliferation, supporting nascent capillary ingrowth into the fibrin clot and restoration of local perfusion (Yakovlev et al., 2014). By day 5 after injury, fibroblasts recruited to the wound edge proliferate and upregulate integrin expression under FG stimulation, facilitating wound-bed infiltration (Suter et al., 2021). The topology of the fibrin network strongly influences fibroblast behavior, including phenotypic transition into myofibroblasts and assembly of actin bundles required for wound contraction (Yuan et al., 2018).

FG-mediated immune modulation contributes to high-quality healing. Physiological fibrin networks limit excessive neutrophil infiltration while enriching non-classical monocytes (Ly6C^low) and pro-resolving macrophages (CD206+ and CX3CR1+). FG can promote macrophage polarization from a pro-inflammatory M1-like phenotype toward an anti-inflammatory M2-like phenotype. *In vivo*, fibrin hydrogels downregulate pro-inflammatory cytokines (IL-6, IL-1β, TNF-α) and upregulate anti-inflammatory IL-10, supporting expansion of dermal endothelial cells, fibroblasts, and keratinocyte re-epithelialization (Pereira et al., 2023). In functionalized systems such as FG-loaded coaxial nanofiber scaffolds, FG enhances paracrine effects of adipose-derived mesenchymal stromal cells (ASCs) and promotes reparative macrophage polarization, helping establish an immunomodulatory niche for skin regeneration (Sun et al., 2021). During maturation, MMPs and the fibrinolytic system degrade the provisional fibrin scaffold. This controlled clearance creates space for deposition of a mature, collagen-rich ECM and restoration of tissue mechanical integrity.

## 4 Pathological extracellular matrix remodeling

In pathological microenvironments such as tumors, fibrosis, and chronic inflammation, FG-ECM interactions undergo substantial alterations. FG’s pathological activity is not necessarily an inherent property of the molecule but may reflect aberrant prolongation of regenerative programs driven by microenvironmental cues such as persistent inflammation, mechanical stress, and proteolytic imbalance. Clarifying this program switch is important for developing selective interventions.

### 4.1 Synergistic pathogenicity of plasma and endogenous FG in tumors

Within the tumor microenvironment (TME), aberrant FG deposition can establish a provisional matrix scaffold. By co-depositing with adhesive glycoproteins, FG sequesters growth factors, recruits immune cells, and promotes angiogenesis, thereby supporting tumor cell adhesion, proliferation, and migration (Simpson-Haidaris and Rybarczyk, 2001). In addition to these established roles, FG and associated family members have recently been characterized as mediators of immunosuppressive networks and inflammatory angiogenesis within the pre-metastatic niche (PMN) (Zhang et al., 2025). Building on these cellular interaction models, the present framework emphasizes structural reorganization of the FG-ECM network. Beyond plasma extravasation, diverse malignant lineages, including lung (A549), hepatic (HepG2), breast (MCF-7), and cervical (ME-180) cancer cells, can synthesize and secrete endogenous FG through upregulated FGA, FGB, and FGG transcription (Sahni et al., 2008). Both plasma-derived and tumor-endogenous FG contribute to TME dynamics. FG interacts with fibroblast growth factor-2 (FGF-2) to amplify proliferative signals and engages integrin αvβ3 to enhance tumor cell adhesion and migration (Sahni et al., 2008). In glioblastoma (GBM), hyperpermeable vasculature facilitates plasma FG deposition, creating a niche that promotes spheroid formation, adhesion, and invasiveness of brain tumor-initiating cells (BTICs). FG can also potentiate matrix metalloproteinase (MMP) activity, facilitating basement membrane degradation and subsequent tumor invasion.

FG also supports the survival and sustained adhesion of circulating metastatic emboli. By engaging intercellular adhesion molecule-1 (ICAM-1) on cancer cells, FG promotes transendothelial migration and extravasation (Liu et al., 2025). In lung carcinoma, the FGG-ICAM-1 axis protects tumor cells from apoptosis and supports tumor progression (Wang et al., 2024). FG has also been reported to upregulate epithelial-mesenchymal transition (EMT) transcription factors, such as Slug and ZEB1, in hepatocellular carcinoma (HCC) metastasis. Consistent with a role for endogenous FG, RNA interference (RNAi)-mediated silencing in prostate cancer models reduces tumor cell proliferation.

As a plastic stromal regulator, FG frequently co-deposits with fibronectin (FN) across solid tumors, forming a provisional matrix rich in integrin ligands that can shield tumors from immune clearance and therapy (Simpson-Haidaris and Rybarczyk, 2001; Wu et al., 2024). The two FG sources, plasma extravasation and local synthesis, may fulfill different roles (Simpson-Haidaris and Rybarczyk, 2001; Wu et al., 2024; Sahni et al., 2008). Plasma-derived FG primarily reflects vascular hyperpermeability and coagulation-inflammation coupling, whereas tumor-endogenous FG may sustain cell-autonomous autocrine or paracrine survival loops (Sahni et al., 2008). Mechanistically, FG contributes to TME progression through three main routes: (1) acting as a scaffold to sequester growth factors such as FGF-2; (2) engaging receptors such as αVβ3 and ICAM-1 to facilitate transendothelial migration and confer anti-apoptotic resistance (Wang et al., 2024); and (3) forming an FN-rich protective niche (Simpson-Haidaris and Rybarczyk, 2001; Wu et al., 2024; El-Ayoubi et al., 2015).

**FIGURE 2:**
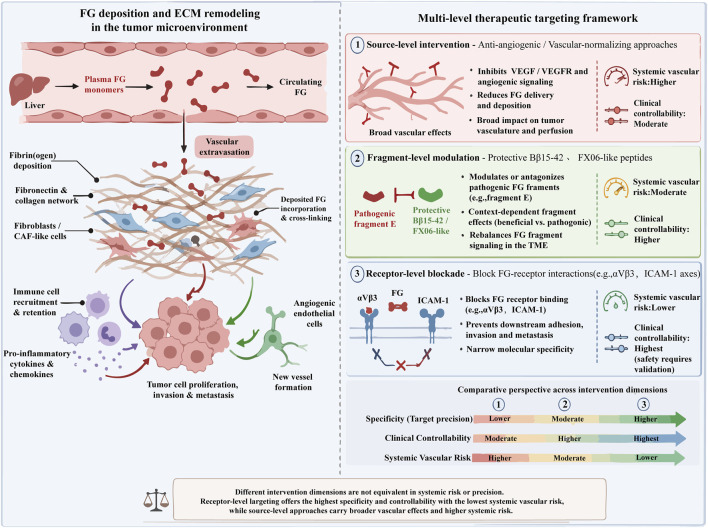
Dual-source FG deposition in the tumor microenvironment and multi-level therapeutic targeting: In the tumor microenvironment, FG accumulates through vascular leakage of plasma-derived FG and, in some tumor types, through local tumor-associated FG production. Once deposited, FG cooperates with FN and other ECM components to form a provisional matrix that promotes tumor cell adhesion, invasion, angiogenesis, immune-cell recruitment, stromal remodeling, and growth factor retention. This dual-source model provides several therapeutic entry points. Source-level strategies, such as vascular normalization or suppression of tumor-associated FG production, may reduce FG accumulation but are more likely to involve broad vascular effects or delivery challenges. Fragment-level modulation aims to limit pathogenic FG-derived signals while preserving protective fragments such as Bβ15-42/FX06-like peptides. Receptor-level blockade focuses on disease-associated FG interactions, including αVβ3 and ICAM-1 axes, and may offer greater molecular specificity, although safety remains to be established. These approaches differ in specificity, systemic risk, and clinical controllability and should be viewed as complementary rather than equivalent strategies.

Unlike the transient matrices in physiological wound healing, this TME-associated FG-FN network can persist aberrantly and maintain the microenvironment in a pathological state. Targeted interventions focused on specific receptor axes (FG-αVβ3, FG-ICAM-1) and tumor-endogenous synthesis may offer greater specificity than global FG depletion. Distinguishing these FG sources has important clinical implications: tumor-endogenous FG may sustain autocrine or paracrine survival loops, whereas plasma-derived FG more closely reflects vascular leakage and coagulation-inflammation coupling. On this basis, we propose a multilevel therapeutic targeting framework (Figure 2). At the source level, strategies aimed at limiting plasma FG extravasation, such as anti-angiogenic or vascular-normalizing approaches, may reduce provisional matrix formation but are likely to have broad vascular effects. By contrast, suppressing tumor-endogenous FG production, for example, through gene-silencing approaches, may provide greater source specificity but requires efficient tumor-selective delivery. At the fragment level, protective FG-derived peptides such as Bβ15-42/FX06-like agents may be used to counteract barrier-disruptive or pro-inflammatory fragment activity, although fragment-specific effects remain context-dependent. At the receptor level, selective blockade of FG-associated receptor axes such as αVβ3 or ICAM-1 may offer narrower molecular specificity than source-level vascular interventions, but hemostatic and immune safety must still be carefully evaluated. These dimensions should therefore be viewed as complementary therapeutic entry points with different degrees of specificity, systemic risk, and clinical controllability.

### 4.2 Inflammation: dual regulatory roles and targeting strategies

Controlled FG deposition and clearance help maintain vascular integrity and initiate acute and reparative inflammatory pathways. In inflammatory niches, fibrin(ogen) modulates cytokine and chemoattractant production, facilitating immune-cell recruitment and subsequent inflammatory amplification. However, unresolved inflammatory activation leads to tissue dysfunction. While physiological FG turnover promotes wound healing in conditions such as inflammatory bowel disease (IBD) (Seltana et al., 2022), impaired fibrinolysis leads to pathological fibrin accumulation, exacerbating inflammation and precipitating thromboembolic events (Davalos and Akassoglou, 2012).

The microenvironmental context shapes FG’s divergent effects. In IBD and DSS-induced colitis models, excessive fibrin(ogen) accumulation subverts its reparative function. Mechanistically, surplus FG disrupts the intestinal barrier and increases vascular permeability by inducing Akt activation, which subsequently promotes epithelial microfilament depolymerization (Zhang et al., 2021). Fibrin(ogen) also contributes to colitis pathophysiology by upregulating cyclooxygenase-2 (COX-2) and stimulating reactive oxygen species (ROS) production. In atherosclerosis, FG deposits on damaged endothelium and complexes with oxidized low-density lipoprotein (oxLDL), accelerating macrophage phagocytosis and foam-cell formation. Subsequent FG accumulation within the vessel wall induces vascular smooth muscle cell (VSMC) proliferation and migration, modulating plaque stability and disease progression. During infection, FG has dual roles. FG deposition can restrict bacterial dissemination and promote clearance in *Staphylococcus aureus* peritonitis, whereas certain pathogens, such as SARS-CoV-2, *Streptococcus*, and *Escherichia coli*, exploit FG through surface-binding proteins to facilitate host dissemination (Wolberg, 2023).

In oral inflammation, FG-neutrophil interactions emerge as a therapeutic target for periodontal disease. In plasminogen-deficient models, impaired fibrinolysis causes fibrin accumulation, which hyperactivates neutrophils. While neutrophils classically capture pathogens via ROS and neutrophil extracellular traps (NETs) (Mo et al., 2024), this uncontrolled fibrin-driven activation induces severe mucosal immunopathology.

#### 4.2.1 Neuroinflammation: fibrin-driven microglial activation and cryptic-epitope targeting

Neuroinflammation provides a clinically important example of pathological FG/fibrin functional switching. In Alzheimer’s disease (AD), traumatic brain injury (TBI), and related neuroinflammatory settings, blood–brain barrier disruption permits plasma FG to enter the CNS parenchyma, where it is converted to fibrin and incorporated into a persistent inflammatory matrix. Extravascular fibrin can promote oxidative stress, inflammatory cytokine release, and neuronal injury, thereby linking vascular leakage to neurodegenerative remodeling (Kantor et al., 2025; Akassoglou, 2024; Guan et al., 2025).

Mechanistically, fibrin can amplify neuroinflammation by exposing cryptic inflammatory epitopes, such as the γ377-395 region, which engages CD11b/CD18 integrin receptors on microglia, also known as αMβ2 or Mac-1. Selective blockade of this inflammatory fibrin epitope, or disruption of fibrin-CD11b interactions, reduces microglial activation and neuroinflammatory injury in experimental models while preserving hemostatic fibrin functions (Adams et al., 2007). More recent fibrin-targeting immunotherapy further indicates that pathogenic fibrin epitopes can be blocked to reduce neuroinflammation and neurodegeneration in experimental models (Ryu et al., 2018). FG inflammatory activity is therefore context-dependent: it supports acute defense and repair through scaffold formation and immune recruitment, but can amplify pathology when deposition persists or receptor engagement becomes aberrant (Yakovlev et al., 2022; Davalos and Akassoglou, 2012). Anti-inflammatory strategies may therefore need to move from indiscriminate FG depletion toward targeted blockade of pathogenic modules. Pathologically, FG/fibrin can promote leukocyte adhesion and inflammatory signaling mainly through αMβ2/Mac-1 and ICAM-1 (Yakovlev et al., 2022; Ugarova and Yakubenko, 2001). It mediates endothelial activation and barrier disruption in atherosclerosis and neuroinflammation (Davalos and Akassoglou, 2012; Ameziane et al., 2003; Kiechl et al., 2002; Sulimai et al., 2021). Conversely, specific FG-derived fragments, such as Bβ15-42/FX06, stabilize the endothelial barrier and attenuate leakage, indicating potentially exploitable protective modules (Bach et al., 1998; Gröger et al., 2009; Wu et al., 2024; Wang et al., 2024; Davalos and Akassoglou, 2012; Atar et al., 2009; Guérin et al., 2023).

Selective reprogramming of FG signaling could represent an important translational direction. Priority interventions include (1) Mac-1/FG interface inhibitors and, where direct evidence supports it, inhibitors of FG-associated TLR4-related inflammatory signaling; (2) localized AKT pathway inhibitors using hydrogel-based sustained-release systems, such as MK-2206, to reduce systemic toxicity; and (3) optimized FX06 analogs with structural modifications that extend half-life. These protective-blockade strategies are designed to preserve physiological healing while suppressing pathological inflammation, but clinical translation requires rigorous safety and efficacy profiling.

### 4.3 Fibrosis: extrahepatic FG as a working example and translational implications

Fibrosis, characterized by excessive ECM accumulation after chronic inflammation, represents a severe pathological endpoint. Through diverse signaling pathways, FG activates fibroblasts and contributes to aberrant matrix deposition, ultimately causing organ dysfunction. Hepatic fibrosis provides a useful example in which liver sinusoidal endothelial cell (LSEC)-derived FG illustrates the translational significance of source heterogeneity. In related fibrotic models, FG acts synergistically with transforming growth factor-β1 (TGF-β1) to induce fibroblast proliferation and activate the TGF-β1/pSMAD2 axis, thereby promoting renal fibrosis (Craciun et al., 2014). Conversely, in hepatic endothelial cells, protein O-fucosyltransferase 1 (POFUT1) attenuates injury-induced fibrosis by repressing FG synthesis (He et al., 2024).

Unlike passive plasma extravasation, stress-induced ectopic FG synthesis by LSECs is uncoupled from the systemic coagulation cascade. It may instead function as a pathological paracrine signal that activates hepatic stellate cells (HSCs) and promotes fibrogenesis. Selectively inhibiting this locally synthesized extrahepatic FG could provide anti-fibrotic efficacy without compromising systemic hemostasis, although its relevance across other organs requires validation. Although the POFUT1-Notch-FG regulatory axis is promising, clinical translation would require highly LSEC-specific nanodelivery systems or antibody-drug conjugates (ADCs) to reprogram endothelial FG synthesis while limiting off-target effects.

Beyond the liver, FG deficiency (Fib−/−) attenuates radiation-induced vascular sclerosis and intestinal wall fibrosis (Wang et al., 2017). In oral submucous fibrosis (OSF), a chronic disorder marked by submucosal collagen deposition (Chiang et al., 2018), FG impairs mucosal repair by disrupting endothelial angiogenesis. FG may also promote epithelial-mesenchymal transition (EMT) of oral mucosa through the TGF-β pathway while increasing tissue inhibitor of metalloproteinases-1 (TIMP-1) expression and thereby suppressing MMP-mediated collagen degradation. Emerging evidence links elevated FG expression to malignant transformation of OSF, potentially through oncogene upregulation or tumor-suppressor inhibition.

In theory, strategies targeting extrahepatic FG in hepatic fibrosis could pursue three avenues: (1) gene therapy driven by LSEC-specific promoters; (2) liver-specific antisense oligonucleotides (ASOs) targeting FGA/FGB/FGG mRNA; and (3) targeted delivery systems using LSEC surface markers such as LYVE-1. Although conceptually supported in other hepatic conditions, their specific inhibitory efficacy against local FG synthesis requires rigorous experimental confirmation.

To consolidate the concept of source heterogeneity highlighted across these pathological models, Table 3 compares hepatic (systemic) and extrahepatic (local/ectopic) FG, outlining their synthetic triggers, microenvironmental roles, and implications for targeted translational interventions.

**TABLE 3:** Distinctive characteristics and translational interventions: Hepatic versus extrahepatic fibrinogen.

Comparative dimension	Hepatic (Systemic/Plasma) fibrinogen	Extrahepatic (Local/Ectopic) fibrinogen
Primary Cellular Source	Predominantly hepatocytes; the primary source of circulating fibrinogen (Castell et al., 1989; Grieninger et al., 1978)	Locally synthesized by pulmonary/intestinal epithelial cells and specific malignant lineages; deposits directly into the basement membrane or tumor stroma (Seltana et al., 2022; Lawrence and Simpson-Haidaris, 2004; Simpson-Haidaris and Rybarczyk, 2001; Wu et al., 2024; Sahni et al., 2008; Hamar, 2024; Rybarczyk and Simpson-Haidaris, 2000; Nguyen and Simpson-Haidaris, 2000; Haidaris, 1997)
Synthetic Trigger Signals	Primarily upregulated by IL-6 during acute-phase responses, synergistically amplified by glucocorticoids; IL-1/TNF act as modulators (Castell et al., 1989; Grieninger et al., 1978)	Co-driven by local IL-6, IL-1β, glucocorticoids, infection/injury, and tumor stress; exhibits cell-type-specific regulation (Seltana et al., 2022; Lawrence and Simpson-Haidaris, 2004; Sahni et al., 2008; Hamar, 2024; Nguyen and Simpson-Haidaris, 2000; Haidaris, 1997)
Mode of Microenvironmental Entry	Reaches the lesion via plasma circulation; extravasates and deposits during increased vascular permeability, hemorrhage, or coagulation activation, subsequently converting to fibrin (Jennewein et al., 2011; Castell et al., 1989; Grieninger et al., 1978)	Secreted basolaterally by local cells directly into the ECM; assembles into matrix fibers (often FN-dependent) without entering systemic circulation (Lawrence and Simpson-Haidaris, 2004; Pereira et al., 2002; Simpson-Haidaris and Rybarczyk, 2001; Haidaris, 1997)
Core Biological Functions	Provides systemic hemostatic substrates; acts as an acute-phase protein linking coagulation, inflammation, and tissue repair (Jennewein et al., 2011; Castell et al., 1989; Grieninger et al., 1978)	Serves as a localized provisional matrix and growth factor hub; directly regulates adhesion, migration, angiogenesis, epithelial repair, and tumor invasion (Lawrence and Simpson-Haidaris, 2004; Pereira et al., 2002; Simpson-Haidaris and Rybarczyk, 2001; Wu et al., 2024; Sahni et al., 2008; Hamar, 2024; Haidaris, 1997)
Pathological Role	Hyperfibrinogenemia and plasma-derived deposition correlate with thrombosis, vascular inflammation, ischemia-reperfusion injury, and neuroinflammation/scarring (Gröger et al., 2009; Jennewein et al., 2011; Petzelbauer et al., 2005; Flick et al., 2004; Adams et al., 2007; Ryu et al., 2018)	Directly orchestrates the lesional microenvironment; exerts sustained effects in pulmonary/intestinal mucosal repair, tumor stroma remodeling, and localized chronic inflammation (Seltana et al., 2022; Lawrence and Simpson-Haidaris, 2004; Simpson-Haidaris and Rybarczyk, 2001; Wu et al., 2024; Sahni et al., 2008; Hamar, 2024; Rybarczyk and Simpson-Haidaris, 2000; Nguyen and Simpson-Haidaris, 2000; Haidaris, 1997)
Limitations of Conventional Interventions	Conventional anticoagulation/fibrinolysis reduces fibrin burden but lacks spatial selectivity, confers systemic bleeding risks, and fails to decouple hemostatic from inflammatory signaling (Jennewein et al., 2011; Flick et al., 2004; Adams et al., 2007; Ryu et al., 2018)	Monitoring or targeting only circulating FG overlooks critical lesional events, including local synthesis, ectopic matrix assembly, and cryptic epitope exposure (Lawrence and Simpson-Haidaris, 2004; Pereira et al., 2002; Simpson-Haidaris and Rybarczyk, 2001; Wu et al., 2024; Hamar, 2024)
Novel Targeted Interventions	Prioritizing blockade of pathological receptor axes over global FG depletion (e.g., β15-42/FX06-VE-cadherin axis, γ377-395-Mac-1 axis, and antibodies targeting cryptic epitopes) (Bach et al., 1998; Gröger et al., 2009; Petzelbauer et al., 2005; Flick et al., 2004; Adams et al., 2007; Ryu et al., 2018; Atar et al., 2009)	Inhibiting local synthesis and ECM assembly (e.g., targeting inflammation-driven transcription or FN-dependent deposition) combined with microenvironmental therapies; currently predominates in preclinical/mechanistic stages (Seltana et al., 2022; Bach et al., 1998; Gröger et al., 2009; Petzelbauer et al., 2005; Lawrence and Simpson-Haidaris, 2004; Pereira et al., 2002; Simpson-Haidaris and Rybarczyk, 2001; Wu et al., 2024; Sahni et al., 2008; Hamar, 2024; Rybarczyk and Simpson-Haidaris, 2000; Nguyen and Simpson-Haidaris, 2000; Haidaris, 1997)
Translational Advantages	FX06 has entered human trials, supporting the translational feasibility of preserving basic hemostasis while selectively attenuating inflammatory or leakage signals. (Gröger et al., 2009; Petzelbauer et al., 2005; Atar et al., 2009)	Theoretically offers spatial selectivity and minimized systemic bleeding risks; compatible with tissue biomarkers, imaging, or localized delivery, though clinical evidence trails systemic pathways (Seltana et al., 2022; Lawrence and Simpson-Haidaris, 2004; Ryu et al., 2018; Wu et al., 2024; Sahni et al., 2008; Hamar, 2024; Rybarczyk and Simpson-Haidaris, 2000)

Abbreviations: IL-6, interleukin-6; TNF, tumor necrosis factor; ECM, extracellular matrix; VE-cadherin, vascular endothelial cadherin; FX06, a Bβ15-42-derived therapeutic peptide. Distinguishing these two sources supports a shift from global systemic FG depletion toward targeted interventions that aim to preserve systemic hemostasis while neutralizing localized pathological signaling in specific microenvironments. Evidence boundary: Hepatocytes are well established as the dominant source of circulating FG, whereas extrahepatic FG is supported only in selected epithelial, endothelial, and tumor-associated contexts. Cell-type-specific claims require direct FGA/FGB/FGG transcript and protein validation; macrophage-derived FG, should remain an unresolved possibility unless direct evidence is provided.

## 5 Clinical translation

The translational potential of FG is evaluated across three dimensions: diagnostic biomarkers, targeted therapeutic interventions, and clinical trials.

### 5.1 Diagnostic and stratification biomarkers

Plasma FG and the fibrinogen-to-albumin ratio (FAR) offer prognostic value in diverse malignancies and inflammatory conditions. FAR predicts overall survival in non-small-cell lung cancer (Ma and Wang, 2024) and identifies active inflammatory bowel disease (Chen et al., 2025). Elevated plasma FG also correlates with advanced hepatocellular carcinoma and poor survival (Zhang and Long, 2017). Nevertheless, as an acute-phase reactant, FG is more useful for risk stratification and disease-activity assessment than as an organ-specific diagnostic marker.

### 5.2 Therapeutic targets and targeted interventions

Therapeutically, selective intervention in pathological FG modules may be preferable to global FG reduction. High-priority targets include the FG-αMβ2/Mac-1 inflammatory axis, context-dependent FG-associated inflammatory pathways, the AKT-mediated barrier-disruption cascade in colitis, and organ-specific extrahepatic FG synthesis (Yakovlev et al., 2022; Medcalf and Keragala, 2021; Santos et al., 2013; Davalos and Akassoglou, 2012; Kiechl et al., 2002; Craciun et al., 2014; He et al., 2024; McGettigan and Shah, 2024). Conversely, therapeutic peptides such as Bβ15-42/FX06 use protective barrier-stabilizing signals within the FG system, providing an example of functional mimicry rather than global inhibition (Gröger et al., 2009; Atar et al., 2009; Guérin et al., 2023).

### 5.3 Source-specific targeting and integrated strategies

**FIGURE 3:**
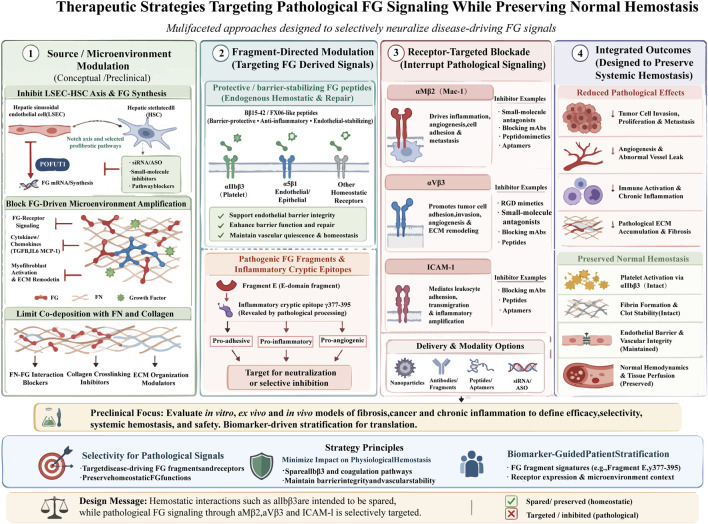
Integrated therapeutic strategies for modulating pathological FG signaling while preserving physiological hemostasis. The figure summarizes complementary intervention levels across pathological FG microenvironments, including source or microenvironment modulation, fragment-directed modulation, receptor-targeted blockade, and downstream pathway or delivery-oriented strategies. Protective Bβ15-42/FX06-like peptides are presented as barrier-stabilizing functional mimics, whereas pathogenic FG-derived fragments or inflammatory cryptic epitopes, such as Fragment E and γ377–395, represent context-dependent targets for selective neutralization. Receptor-level strategies focus on disease-associated FG interactions, including αMβ2/Mac-1, αVβ3, and ICAM-1, while aiming to spare core hemostatic functions such as αIIbβ3-dependent platelet–fibrin interactions. Source- and pathway-level strategies remain largely conceptual or preclinical and require validation in disease-specific delivery systems. Overall, the framework emphasizes selective suppression of pathological FG signaling rather than global FG depletion.

Although inhibitors targeting extrahepatic FG synthesis, such as LSEC-specific POFUT1 activation or FG siRNA, are theoretically viable, clinical translation faces substantial barriers. The strongest current evidence is limited to liver fibrosis, LSEC-specific delivery systems pose unresolved technical challenges, and the potential side effects of broadly inhibiting extrahepatic FG during tissue regeneration remain undefined. Consequently, source-specific targeting remains conceptual. Synthesizing these insights, interventions for pathological FG microenvironments can be organized into four strategies (Figure 3): (1) source inhibition of ectopic non-hepatic FG synthesis; (2) receptor blockade of pro-inflammatory interactions; (3) pathway inhibition targeting downstream cytoskeletal disruption; and (4) functional mimicry using protective peptides to stabilize endothelial barriers.

### 5.4 Clinical trials and early translational evidence

The most advanced clinical candidate targeting the FG axis is FX06, a Bβ15-42-derived peptide. It has completed the F.I.R.E. trial for ST-segment elevation myocardial infarction (STEMI) reperfusion injury and has advanced to trials for COVID-19-associated acute respiratory distress syndrome (ARDS), providing clinical testing of FG fragments as barrier-protective therapeutics (Atar et al., 2009; Guérin et al., 2023; Kloka et al., 2022). Pharmacologically, FX06 stabilizes endothelial junctions, dampens acute inflammation, and attenuates vascular leakage. *Ex vivo* studies indicate that FX06 counteracts SARS-CoV-2-triggered cytokine storms and pulmonary barrier disruption by modulating the RhoA/ROCK1 pathway.

However, clinical efficacy remains context-dependent. For COVID-19-associated ARDS, a small trial (n = 25) evaluated intravenous FX06 in mechanically ventilated patients, focusing on the extravascular lung water index (EVLWi) (Guérin et al., 2023). The subsequent IXION trial (n = 134) in non-intubated patients suggested potential reductions in intubation and mortality rates. These findings require validation in larger clinical cohorts (Polzin et al., 2025; Rao et al., 2022). In the F.I.R.E. STEMI trial (n = 234), FX06 did not meet its primary endpoint of reducing overall infarct size, which remains a major translational limitation for FG-derived therapeutics in cardiovascular reperfusion injury. The reported subgroup benefit in patients treated within 4 h, despite its statistical signal, should be interpreted as hypothesis-generating rather than definitive evidence of efficacy. Future development of FX06-like agents should therefore address not only timing but also pharmacokinetics, target-organ retention, patient selection, endpoint design, and independent validation in adequately powered trials.

Building on these limitations, next-generation optimization of FX06-like agents may prioritize: (1) improving target-organ retention through pharmaceutical modifications, such as conjugation with hyaluronic acid; and (2) developing longer-acting formulations for chronic endothelial dysfunction. These strategies should be evaluated as potential approaches to addressing current translational bottlenecks in endothelial-targeted therapies.

## 6 Translational applications: from tissue engineering to smart biomaterials

Several reviews have summarized the chemistry, fabrication strategies, and tissue-engineering applications of fibrin/FG-based biomaterials, including fibrin hydrogels, sealants, composite scaffolds, and biofunctionalized matrices (Sanz-Horta et al., 2023; Li S. et al., 2024). Rather than repeating this broader biomaterials literature, this section focuses on how the functional-switching, source-heterogeneity, and mechanosensing frameworks developed above can be translated into FG-material design principles. Stiffness, viscoelasticity, ligand presentation, and FG/FN co-assembly correspond to mechanotransduction nodes discussed in Section 2.4; controlled FG recruitment, retention, or local presentation reflects the source-heterogeneity framework; and protease-sensitive degradation links material design to fragment-generation mechanisms discussed in Section 2.3. Thus, FG-based biomaterials should be viewed not simply as passive scaffolds but as programmable microenvironmental systems intended to bias FG-associated signaling toward repair while limiting persistent inflammatory, fibrotic, or tumor-supportive remodeling.

### 6.1 Tissue engineering and regeneration

During tissue healing, FG can act beyond a passive scaffold by guiding cell behavior through localized enrichment of growth factors such as VEGF and TGF-β. Combining FG with polyethylene glycol (PEG), cellulose nanocrystals (CNC), or collagen allows tuning of mechanical strength and degradation kinetics (Goldshmid et al., 2015; Wang et al., 2010). For instance, an FG monolayer on titanium alloy promotes osteogenic differentiation and ECM mineralization by exposing RGD motifs (Shepherd et al., 2017). CNC-reinforced FG nanocomposite hydrogels also show improved injectability and gelation and have been associated with enhanced muscle tissue regeneration (Wang K. et al., 2021).

#### 6.1.1 Biophysical design parameters of FG materials for tissue regeneration

In clinical translation, FG-based biomaterials function as more than biochemical delivery vehicles. Their encoded biophysical properties, including stiffness, topography, and viscoelasticity, can act as instructive parameters that shape tissue-specific regeneration outcomes.

1. Tissue-Specific Stiffness Thresholds: Matching the mechanical microenvironment of the target tissue is a physical prerequisite for guiding cell fate. In neural regeneration, related hydrogel studies indicate that matrix stiffness (e.g., 6.1–110.5 kPa in modified gels) can cooperate with topography to increase the proportion of β-tubulin III (TUJ1)-positive neurons and promote neurite extension (Mattiassi et al., 2023). For soft tissue and dental pulp repair, specialized FG-blood hydrogels and stiffness-matched matrices can recapitulate native tissue viscoelasticity and enhance long-term survival and metabolic activity of resident stem cells (Liu et al., 2024; Piglionico et al., 2023). During osteogenesis, adjusting interfacial stiffness without altering chemical or topographical cues can act as a mechanical switch that controls mesenchymal stem-cell adhesion and stiffness-dependent differentiation (Zanut et al., 2023). Conversely, mechanical mismatch between the scaffold and surrounding tissue impairs regeneration and leads to abnormal development (Xu et al., 2021).
2. Programmed Viscoelasticity and Stress Relaxation: Native biological tissues are dynamically viscoelastic. Stress-relaxation kinetics in FG hydrogels may provide a regulatory axis distinct from static stiffness. Related viscoelastic matrix studies show that increasing extracellular fluid viscosity can reverse adipogenic lineage commitment of human mesenchymal stem cells on low-stiffness substrates (Amitrano et al., 2025). Modifying crosslinking networks to accelerate hydrogel stress relaxation, such as 60% relaxation within 10 min, alters mesh size and can regulate cell fate and drug responses in 3D microenvironments (Zhao et al., 2024; Sinha et al., 2023). Altering viscoelasticity through covalent crosslinking variations alone is sufficient to induce distinct functional phenotypes in T cells receiving identical stimuli (Adu-Berchie et al., 2023). For injectable systems, adjusting FG concentration directly tunes storage and loss moduli together with creep compliance, providing a basis for optimizing shear-thinning performance (Fuenteslopez et al., 2025).
3. Topographical Guidance and Spatial Porosity: Fiber alignment and mesoscopic pore structure influence the directionality of tissue assembly and mass transport. FG nanofibrous scaffolds prepared by salt-induced self-assembly (Young’s modulus ∼1.3 MPa) recapitulate features of native blood clots; they support fibroblast adhesion and migration while exhibiting topographical properties that inhibit *Escherichia coli* growth (Suter et al., 2021). In collagen-fibrin interpenetrating networks and FG composite hydrogels, increased fiber content and altered crosslinking density reduce mesh size and tune macromolecular diffusion, water absorption, and growth-factor release (Choi et al., 2026; Jung et al., 2023). For complex interfacial defects, anisotropic multilayered scaffolds, such as FG-modified decalcified bone matrix, generate elastic modulus gradients up to 174.2 MPa and improve mechanical matching and vascularization in osteochondral regeneration zones (Chen et al., 2022). Incorporating spatiotemporal mechanical cues, such as dynamic stiffness softening, remains important for guiding morphogenesis and developmental trajectories of 3D organoids (Xie et al., 2025).

#### 6.1.2 Translating functional switching and source heterogeneity into FG-material design

The functional-switching framework suggests that FG-based materials should be designed to favor transient, reparative FG signaling rather than persistent pathological matrix signaling (Sanz-Horta et al., 2023; Xie et al., 2025). Mechanically, this requires programming scaffold stiffness and viscoelasticity to approximate the target tissue niche while avoiding excessive stiffening that may promote sustained integrin clustering, myofibroblast activation, or fibrosis-like ECM deposition (Ting et al., 2021; Dalton and Lemmon, 2021; Mattiassi et al., 2023; Zanut et al., 2023; Amitrano et al., 2025; Adu-Berchie et al., 2023; Xie et al., 2025). Because FG-specific stiffness thresholds remain insufficiently defined, stiffness gradients, molecular tension probes, or dynamically tunable hydrogels may help identify mechanical windows that favor repair-associated FG-integrin responses (Ting et al., 2021; Han et al., 2024; Zhang et al., 2023; Sarkar et al., 2023; Mattiassi et al., 2023; Xie et al., 2025).

The source-heterogeneity framework adds a second design layer. Materials should distinguish between passive recruitment of circulating plasma FG, controlled retention of endogenous FG, and local presentation of defined FG-derived peptides or fragments (Seltana et al., 2022; Jennewein et al., 2011; Lawrence and Simpson-Haidaris, 2004; Pereira et al., 2002; Simpson-Haidaris and Rybarczyk, 2001; Sahni et al., 2008; Hamar, 2024; Rybarczyk and Simpson-Haidaris, 2000; Nguyen and Simpson-Haidaris, 2000; Haidaris, 1997). For example, vascularized implants may need to limit uncontrolled plasma FG adsorption and fibrin-rich inflammatory matrix formation, whereas regenerative scaffolds may benefit from controlled presentation of protective FG-derived motifs or fragments (Bach et al., 1998; Gröger et al., 2009; Petzelbauer et al., 2005; Kearney et al., 2022; Atar et al., 2009; Guérin et al., 2023). In parallel, FG/FN co-assembly, αC-domain exposure, FN mechanics, and EDA-FN enrichment may be tuned to regulate ligand presentation and integrin engagement (Kidwai et al., 2016; Pereira et al., 2002; Dalton and Lemmon, 2021; Patten et al., 2025; Li W. et al., 2024; Yakovlev et al., 2014).

Finally, protease-sensitive design links biomaterials to FG fragmentomics. Because FG degradation can generate fragments with divergent biological activities, material degradability should be programmed to control the timing and composition of fragment release (Nencini et al., 2024; Fuchs et al., 2020; Zhang et al., 2024; Fan et al., 2022). This may help preserve protective activities, such as Bβ15-42/FX06-like barrier-stabilizing effects, while limiting accumulation of pro-inflammatory or pro-angiogenic degradation products (Bootle-Wilbraham et al., 2001; Gröger et al., 2009; Jennewein et al., 2011; Lee et al., 1999; Fuchs et al., 2020; Davalos and Akassoglou, 2012). Together, these principles translate FG functional switching from a descriptive biological framework into experimentally testable material design rules.

### 6.2 Extrahepatic FG regulation of pathological microenvironments

In pathological contexts, modulating FG crosslinking density or introducing sulfation modifications may help control macrophage polarization and metabolic intervention. Controllable gelation approaches may also physically restrict tumor nutrient supply and induce metabolic stress in cancer cells (Zheng et al., 2020). In addition, FG-based hydrogel microspheres may enrich the plasminogen system and modulate fibrinolysis-coagulation balance, thereby suppressing fibrotic responses. However, static FG materials may be poorly suited to dynamic disease evolution; spatiotemporal intervention will likely require materials that sense and respond to specific microenvironmental cues.

#### 6.2.1 Stimulus-responsive biomaterials

Stimulus-responsive biomaterials that sense pathological cues, such as MMP overexpression, acidic pH, and ROS accumulation, may provide a route for controlled degradation and on-demand release.

1. MMP-Responsive FG Carriers: Introducing MMP-cleavable substrates into the FG network could enable on-demand degradation in high-MMP environments, analogous to the use of such sequences in PEG/gelatin hydrogels for targeted drug release (Zhang et al., 2024; Fan et al., 2022).
2. pH- and ROS-Responsive FG Scaffolds: Inspired by analogous pH-sensitive and redox-responsive gelatin or silk systems (Zheng et al., 2025; Wang Y. et al., 2021; Lin et al., 2024; Zuo et al., 2026), pH/ROS-responsive FG nanocarriers could use acidic tumor microenvironments or high-ROS wound beds to trigger molecular unfolding. This conformational shift may expose cryptic cell-adhesion motifs and enhance targeted cellular uptake, but FG-specific validation remains needed.

**FIGURE 4:**
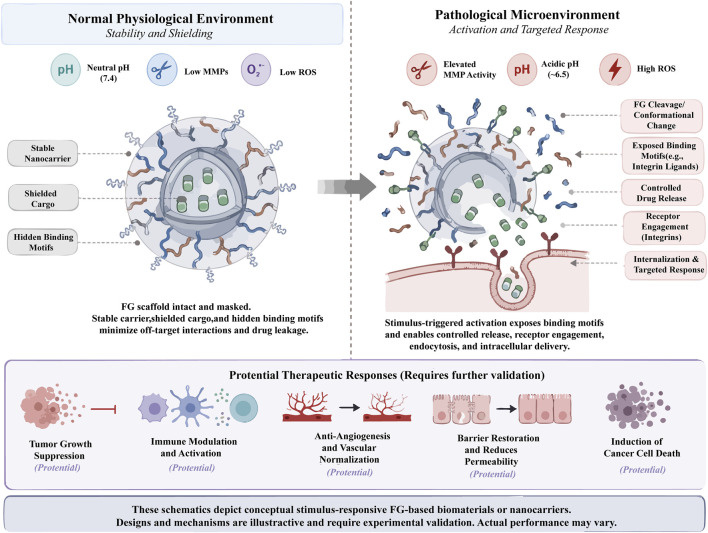
Conceptual design of stimulus-responsive FG-based biomaterials: FG-based biomaterials can be engineered to remain stable under physiological conditions and respond selectively to disease-associated cues. Under neutral pH, low MMP activity, and limited oxidative stress, the carrier is designed to protect encapsulated cargo and keep adhesive or targeting motifs concealed. In pathological microenvironments, elevated MMP activity, acidic pH, or increased ROS may trigger scaffold cleavage, conformational changes, exposure of binding motifs, and controlled cargo release. This design concept extends the functional-switching logic of FG to biomaterials: the same FG-derived structure may support repair or contribute to pathology depending on how it is presented, degraded, and recognized by surrounding cells. Although stimulus-responsive FG-based platforms may improve localized delivery and microenvironment-specific intervention, their FG-specific degradation profiles, fragment generation, receptor engagement, and *in vivo* safety require further validation.

As illustrated in Figure 4, under physiological conditions, the smart FG nanocarrier is designed to maintain structural stability, shield encapsulated drugs, and conceal targeting motifs. After entering a pathological niche, the carrier network may undergo cue-specific cleavage or conformational unfolding. This environment-triggered mechanism is intended to support on-demand drug release and expose integrin-binding motifs, thereby enhancing receptor-mediated endocytosis.

### 6.3 Translational limitations and future directions

Despite their promise, FG-based biomaterials face several translational barriers. First, because FG is intrinsically linked to coagulation, fibrinolysis, and inflammation, uncontrolled FG presentation may increase thrombotic, inflammatory, or fibrotic risk (Yakovlev et al., 2022; Pechlivani et al., 2021; Nencini et al., 2024; Jennewein et al., 2011; Davalos and Akassoglou, 2012). Second, material performance may vary with FG source, purification method, crosslinking density, viscoelasticity, and degradation kinetics, which can affect reproducibility and regulatory translation (Sanz-Horta et al., 2023; Li S. et al., 2024; Piglionico et al., 2023). Third, proteolytic degradation can generate mixtures of fragments with divergent biological effects; therefore, scaffold degradation should be evaluated not only by mass loss or mechanical weakening, but also by fragment identity and function (Nencini et al., 2024; Bootle-Wilbraham et al., 2001; Gröger et al., 2009; Jennewein et al., 2011; Lee et al., 1999). Finally, most current FG-based or fibrin-based biomaterial platforms remain validated mainly *in vitro* or in small-animal models, and causal links between defined material parameters and FG-specific receptor engagement *in vivo* remain incompletely established (Sanz-Horta et al., 2023; Li S. et al., 2024). Future platforms should therefore combine tunable mechanics, controlled proteolysis, spatial proteomics, and FG fragmentomics to define safer design windows for regenerative and disease-targeted applications (Ting et al., 2021; Sanz-Horta et al., 2023; Li S. et al., 2024; Zhang et al., 2024).

## 7 Conclusions and outlook

Fibrinogen (FG) is increasingly recognized not only as a terminal substrate of the coagulation cascade but also as a context-dependent regulator of extracellular matrix (ECM) remodeling. This review integrates these functions into two working frameworks: a microenvironmental functional switch, in which localized proteolytic fragments and receptor specificities shape biological outcomes, and source heterogeneity, which distinguishes systemic plasma FG from locally synthesized or ectopically deposited FG within pathological niches.

This perspective shifts therapeutic thinking from global FG depletion toward selective interventions. By identifying localized FG pathways, such as ectopic synthesis or specific pathological receptor axes, it may be possible to suppress disease progression while preserving systemic hemostasis, although this goal remains to be tested in disease-specific models and clinical settings.

Future research should prioritize FG fragmentomics to identify disease-specific proteolytic profiles and should further clarify how mechanotransduction influences functional switching. Stimulus-responsive biomaterials that use these dynamic mechanisms may support next-generation regenerative strategies. Progress will depend on linking molecular insights to organ-specific targeting and on validating FG-directed approaches in appropriately powered preclinical and clinical studies.

## References

[B1] Adams R. A. Bauer J. Flick M. J. Sikorski S. L. Nuriel T. Lassmann H. (2007). The fibrin-derived gamma377-395 peptide inhibits microglia activation and suppresses relapsing paralysis in central nervous system autoimmune disease. J. Exp. Med. 204, 571–582. 10.1084/jem.20061931 17339406 PMC2137908

[B2] Adu-Berchie K. Liu Y. Zhang D. K. Y. Freedman B. R. Brockman J. M. Vining K. H. (2023). Generation of functionally distinct T-cell populations by altering the viscoelasticity of their extracellular matrix. Nat. Biomed. Eng. 7, 1374–1391. 10.1038/s41551-023-01052-y 37365267 PMC10749992

[B3] Akassoglou K. (2024). Unlocking blood drivers of neurodegeneration: Mechanisms and therapies. Alzheimers Dement. 20, e083655. 10.1002/alz.083655

[B4] Ameziane N. Beillat T. Verpillat P. Chollet-Martin S. Aumont M. C. Seknadji P. (2003). Association of the toll-like receptor 4 gene Asp299Gly polymorphism with acute coronary events. Arterioscler. Thromb. Vasc. Biol. 23, e61–e64. 10.1161/01.ATV.0000101191.92392.1D 14563652

[B5] Amitrano A. Yuan Q. Agarwal B. Sen A. Dance Y. W. Zuo Y. (2025). Extracellular fluid viscosity regulates human mesenchymal stem cell lineage and function. Sci. Adv. 11, eadr5023. 10.1126/sciadv.adr5023 39742493 PMC11691697

[B6] Aran D. Looney A. P. Liu L. Wu E. Fong V. Hsu A. (2019). Reference-based analysis of lung single-cell sequencing reveals a transitional profibrotic macrophage. Nat. Immunol. 20, 163–172. 10.1038/s41590-018-0276-y 30643263 PMC6340744

[B7] Arulmoli J. Wright H. J. Phan D. T. T. Sheth U. Que R. A. Botten G. A. (2016). Combination scaffolds of salmon fibrin, hyaluronic acid, and laminin for human neural stem cell and vascular tissue engineering. Acta Biomater. 43, 122–138. 10.1016/j.actbio.2016.07.043 27475528 PMC5386322

[B8] Atar D. Petzelbauer P. Schwitter J. Huber K. Rensing B. Kasprzak J. D. (2009). Effect of intravenous FX06 as an adjunct to primary percutaneous coronary intervention for acute ST-segment elevation myocardial infarction results of the F.I.R.E. (efficacy of FX06 in the prevention of myocardial reperfusion injury) trial. J. Am. Coll. Cardiol. 53, 720–729. 10.1016/j.jacc.2008.12.017 19232907

[B9] Bach T. L. Barsigian C. Yaen C. H. Martinez J. (1998). Endothelial cell VE-cadherin functions as a receptor for the beta15-42 sequence of fibrin. J. Biol. Chem. 273, 30719–30728. 10.1074/jbc.273.46.30719 9804847

[B10] Becatti M. Emmi G. Bettiol A. Mannucci A. Argento F. R. Fini E. (2026). Reactive oxygen species-induced modifications of fibrin clots as a link between immune responses and atherothrombosis in systemic lupus erythematosus. Arthritis Rheumatol. 78, 344–356. 10.1002/art.43371 40897511 PMC12936896

[B11] Bini A. Itoh Y. Kudryk B. J. Nagase H. (1996). Degradation of cross-linked fibrin by matrix metalloproteinase 3 (stromelysin 1): hydrolysis of the γ gly 404−Ala 405 peptide bond. Biochemistry 35, 13056–13063. 10.1021/bi960730c 8855941

[B12] Bini A. Wu D. Schnuer J. Kudryk B. J. (1999). Characterization of stromelysin 1 (MMP-3), matrilysin (MMP-7), and membrane type 1 matrix metalloproteinase (MT1-MMP) derived fibrin(ogen) fragments D-Dimer and D-like monomer: NH2-terminal sequences of late-stage digest fragments. Biochemistry 38, 13928–13936. 10.1021/bi991096g 10529239

[B13] Bonadio J. D. Bashiri G. Halligan P. Kegel M. Ahmed F. Wang K. (2024). Delivery technologies for therapeutic targeting of fibronectin in autoimmunity and fibrosis applications. Adv. Drug Deliv. Rev. 209, 115303. 10.1016/j.addr.2024.115303 38588958 PMC11111362

[B14] Bonnans C. Chou J. Werb Z. (2014). Remodelling the extracellular matrix in development and disease. Nat. Rev. Mol. Cell Biol. 15, 786–801. 10.1038/nrm3904 25415508 PMC4316204

[B15] Bootle-Wilbraham C. A. Tazzyman S. Thompson W. D. Stirk C. M. Lewis C. E. (2001). Fibrin fragment E stimulates the proliferation, migration and differentiation of human microvascular endothelial cells *in vitro* . Angiogenesis 4, 269–275. 10.1023/a:1016076121918 12197472

[B16] Budzynski A. Z. Marder V. J. (1977). Degradation pathway of fibrinogen by plasmin. Thromb. Haemost. 38, 793–800. 10.1055/s-0038-1651898 146273

[B17] Bugatti S. De Stefano L. Gandolfo S. Ciccia F. Montecucco C. (2023). Autoantibody-negative rheumatoid arthritis: still a challenge for the rheumatologist. Lancet Rheumatol. 5, e743–e755. 10.1016/s2665-9913(23)00242-4 38251565

[B18] Bustos A. H. Brüner M. Kragstrup T. W. Astakhova K. (2025). Citrullinated peptides as drug candidates for rheumatoid arthritis. Front. Immunol. 16, 1648913. 10.3389/fimmu.2025.1648913 41376634 PMC12685853

[B19] Castell J. V. Gómez-Lechón M. J. David M. Andus T. Geiger T. Trullenque R. (1989). Interleukin-6 is the major regulator of acute phase protein synthesis in adult human hepatocytes. FEBS Lett. 242, 237–239. 10.1016/0014-5793(89)80476-4 2464504

[B20] Chapin J. C. Hajjar K. A. (2015). Fibrinolysis and the control of blood coagulation. Blood Rev. 29, 17–24. 10.1016/j.blre.2014.09.003 25294122 PMC4314363

[B21] Chen C. S. Chou S. H. Thiagarajan P. (1988). Fibrin(ogen) peptide B beta 15-42 inhibits platelet aggregation and fibrinogen binding to activated platelets. Biochemistry 27, 6121–6126. 10.1021/bi00416a044 3191112

[B22] Chen Z. Du W. Lv Y. (2022). Zonally stratified decalcified bone scaffold with different stiffness modified by fibrinogen for osteochondral regeneration of knee joint defect. ACS Biomater. Sci. Eng. 8, 5257–5272. 10.1021/acsbiomaterials.2c00813 36335510

[B23] Chen Y. Li Z. Kong F. Ju L. A. Zhu C. (2024). Force-regulated spontaneous conformational changes of integrins alpha(5)beta(1) and alpha(V)beta(3). ACS Nano 18, 299–313. 10.1021/acsnano.3c06253 38105535 PMC10786158

[B24] Chen X. F. Huang Z. M. Huang X. L. (2025). Fibrinogen-to-albumin ratio: a new biomarker to identify inflammatory bowel disease in active stage. Front. Med. (Lausanne) 12, 1460975. 10.3389/fmed.2025.1460975 40046919 PMC11879998

[B25] Chiang C. P. Yu-Fong Chang J. Wu Y. H. Sun A. Wang Y. P. Chen H. M. (2018). Hematinic deficiencies and anemia in gastric parietal cell antibody-positive and -negative oral submucous fibrosis patients. J. Dent. Sci. 13, 68–74. 10.1016/j.jds.2018.02.001 30895097 PMC6388861

[B26] Choi S. R. Mellican S. M. Roberts T. J. Kang S. Elzey B. D. Kong H. (2026). Structure-functionality relationship of collagen-fibrin interpenetrating hydrogels for engineered tumor-stroma models. Acta Biomater. 214, 271–284. 10.1016/j.actbio.2026.03.023 41825815 PMC13235778

[B27] Craciun F. L. Ajay A. K. Hoffmann D. Saikumar J. Fabian S. L. Bijol V. (2014). Pharmacological and genetic depletion of fibrinogen protects from kidney fibrosis. Am. J. Physiol. Ren. Physiol. 307, F471–F484. 10.1152/ajprenal.00189.2014 25007874 PMC4137131

[B28] Dalton C. J. Lemmon C. A. (2021). Fibronectin: molecular structure, fibrillar structure and mechanochemical signaling. Cells 10, 2443. 10.3390/cells10092443 34572092 PMC8471655

[B29] Davalos D. Akassoglou K. (2012). Fibrinogen as a key regulator of inflammation in disease. Semin. Immunopathol. 34, 43–62. 10.1007/s00281-011-0290-8 22037947

[B30] Di Russo J. Young J. L. Wegner J. W. Steins T. Kessler H. Spatz J. P. (2021). Integrin alpha5beta1 nano-presentation regulates collective keratinocyte migration independent of substrate rigidity. Elife 10, e69861. 10.7554/eLife.69861 34554089 PMC8460267

[B31] Dobson D. A. Fish R. J. de Vries P. S. Morrison A. C. Neerman-Arbez M. Wolberg A. S. (2024). Regulation of fibrinogen synthesis. Thromb. Res. 242, 109134. 10.1016/j.thromres.2024.109134 39216273 PMC11381137

[B32] Duong H. Wu B. Tawil B. (2009). Modulation of 3D fibrin matrix stiffness by intrinsic fibrinogen-thrombin compositions and by extrinsic cellular activity. Tissue Eng. Part A 15, 1865–1876. 10.1089/ten.tea.2008.0319 19309239 PMC2749875

[B33] El-Ayoubi F. Amiral J. Pascaud J. Charrin S. Tassel B. Uzan G. (2015). A fibrin antibody binding to fibronectin induces potent inhibition of angiogenesis. Thromb. Haemost. 113, 143–153. 10.1160/TH14-01-0020 25252851

[B34] Erb E. M. Tangemann K. Bohrmann B. Müller B. Engel J. (1997). Integrin alphaIIb beta3 reconstituted into lipid bilayers is nonclustered in its activated state but clusters after fibrinogen binding. Biochemistry 36, 7395–7402. 10.1021/bi9702187 9200686

[B35] Fan C. Yang W. Zhang L. Cai H. Zhuang Y. Chen Y. (2022). Restoration of spinal cord biophysical microenvironment for enhancing tissue repair by injury-responsive smart hydrogel. Biomaterials 288, 121689. 10.1016/j.biomaterials.2022.121689 35931574

[B36] Feng J. Feng K. Xiong Z. Yu M. Hu Y. Wang W. (2025). Capped high-force integrin bond lifetimes and spacing-tuned binding frequency drive rapid fibroblast migration. Proc. Natl. Acad. Sci. U S A. 122, e2505941122. 10.1073/pnas.2505941122 41264256 PMC12663982

[B37] Flick M. J. Du X. Witte D. P. Jirousková M. Soloviev D. A. Busuttil S. J. (2004). Leukocyte engagement of fibrin(ogen) via the integrin receptor alphaMbeta2/Mac-1 is critical for host inflammatory response *in vivo* . J. Clin. Invest 113, 1596–1606. 10.1172/JCI20741 15173886 PMC419487

[B38] Fuchs P. Calitz C. Pavlović N. Binet F. Solbak S. Danielson U. H. (2020). Fibrin fragment E potentiates TGF-β-induced myofibroblast activation and recruitment. Cell Signal 72, 109661. 10.1016/j.cellsig.2020.109661 32334027

[B39] Fuenteslopez C. V. Papapavlou M. Thompson M. S. Ye H. (2025). Engineering a long-lasting microvasculature *in vitro* model for traumatic injury research. Biomater. Adv. 174, 214310. 10.1016/j.bioadv.2025.214310 40220460

[B40] Goldshmid R. Cohen S. Shachaf Y. Kupershmit I. Sarig-Nadir O. Seliktar D. (2015). Steric interference of adhesion supports *in-vitro* chondrogenesis of mesenchymal stem cells on hydrogels for cartilage repair. Sci. Rep. 5, 12607. 10.1038/srep12607 26411496 PMC4585928

[B41] Gómez-Bañuelos E. Konig M. F. Andrade F. (2022). Microbial pathways to subvert host immunity generate citrullinated neoantigens targeted in rheumatoid arthritis. Curr. Opin. Struct. Biol. 75, 102423. 10.1016/j.sbi.2022.102423 35834948 PMC9668488

[B42] Grieninger G. Hertzberg K. M. Pindyck J. (1978). Fibrinogen synthesis in serum-free hepatocyte cultures: stimulation by glucocorticoids. Proc. Natl. Acad. Sci. U S A. 75, 5506–5510. 10.1073/pnas.75.11.5506 281699 PMC392994

[B43] Groeneveld D. Pereyra D. Veldhuis Z. Adelmeijer J. Ottens P. Kopec A. K. (2019). Intrahepatic fibrin(ogen) deposition drives liver regeneration after partial hepatectomy in mice and humans. Blood 133, 1245–1256. 10.1182/blood-2018-08-869057 30655274 PMC6418476

[B44] Gröger M. Pasteiner W. Ignatyev G. Matt U. Knapp S. Atrasheuskaya A. (2009). Peptide Bbeta(15-42) preserves endothelial barrier function in shock. PLoS One 4, e5391. 10.1371/journal.pone.0005391 19401765 PMC2670535

[B45] Guan S. Lu T. Li L. Zhang Y. Huang C. Li C. (2025). Targeting hypercoagulability in ischemic stroke modulates fibrin-driven microglial polarization via JAK-STAT pathway. J. Neuroinflammation 22, 258. 10.1186/s12974-025-03582-5 41188981 PMC12584507

[B46] Guérin E. Belin L. Franchineau G. Le Guennec L. Hajage D. Diallo M. H. (2023). FX06 to rescue SARS-CoV-2-induced acute respiratory distress syndrome: a randomized clinical trial. Crit. Care 27, 331. 10.1186/s13054-023-04616-1 37641136 PMC10463389

[B47] Haidaris P. J. (1997). Induction of fibrinogen biosynthesis and secretion from cultured pulmonary epithelial cells. Blood 89, 873–882. 10.1182/blood.V89.3.873 9028318

[B48] Hamaguchi M. Bunce L. A. Sporn L. A. Francis C. W. (1993). Spreading of platelets on fibrin is mediated by the amino terminus of the beta chain including peptide beta 15-42. Blood 81, 2348–2356. 10.1182/blood.V81.9.2348.2348 8481515

[B49] Hamar P. (2024). Local production of acute phase proteins: a defense reaction of cancer cells to injury with focus on fibrinogen. Int. J. Mol. Sci. 25, 3435. 10.3390/ijms25063435 38542407 PMC10970835

[B50] Han S. B. Lee G. Kim D. Kim J. K. Kim I. S. Kim H. W. (2024). Selective suppression of integrin-ligand binding by single molecular tension probes mediates directional cell migration. Adv. Sci. (Weinh) 11, e2306497. 10.1002/advs.202306497 38311584 PMC11005741

[B51] Hantgan R. R. Stahle M. C. Lord S. T. (2010). Dynamic regulation of fibrinogen: integrin αIIbβ3 binding. Biochemistry 49, 9217–9225. 10.1021/bi1009858 20828133 PMC3210020

[B52] Hastings J. F. Skhinas J. N. Fey D. Croucher D. R. Cox T. R. (2019). The extracellular matrix as a key regulator of intracellular signalling networks. Br. J. Pharmacol. 176, 82–92. 10.1111/bph.14195 29510460 PMC6284331

[B53] He S. Luo Y. Ma W. Wang X. Yan C. Hao W. (2024). Endothelial POFUT1 controls injury-induced liver fibrosis by repressing fibrinogen synthesis. J. Hepatol. 81, 135–148. 10.1016/j.jhep.2024.02.032 38460791

[B54] Hiller O. Lichte A. Oberpichler A. Kocourek A. Tschesche H. (2000). Matrix metalloproteinases Collagenase-2, macrophage elastase, Collagenase-3, and membrane type 1-Matrix metalloproteinase impair clotting by degradation of fibrinogen and factor XII. J. Biol. Chem. 275, 33008–33013. 10.1074/jbc.M001836200 10930399

[B55] Horasawa N. Yamashita T. Uehara S. Udagawa N. (2015). High-performance scaffolds on titanium surfaces: osteoblast differentiation and mineralization promoted by a globular fibrinogen layer through cell-autonomous BMP signaling. Mater Sci. Eng. C Mater Biol. Appl. 46, 86–96. 10.1016/j.msec.2014.10.025 25491963

[B56] Hsieh J. Y. Smith T. D. Meli V. S. Tran T. N. Botvinick E. L. Liu W. F. (2017). Differential regulation of macrophage inflammatory activation by fibrin and fibrinogen. Acta Biomater. 47, 14–24. 10.1016/j.actbio.2016.09.024 27662809 PMC5426227

[B57] Hurlet-Jensen A. Koehn J. A. Nossel H. L. (1983). The release of B beta 1-42 from fibrinogen and fibrin by plasmin. Thromb. Res. 29, 609–617. 10.1016/0049-3848(83)90215-3 6222508

[B58] Jennewein C. Tran N. Paulus P. Ellinghaus P. Eble J. A. Zacharowski K. (2011). Novel aspects of fibrin(ogen) fragments during inflammation. Mol. Med. 17, 568–573. 10.2119/molmed.2010.00146 21210072 PMC3105136

[B59] Jo M. H. Li J. Jaumouille V. Hao Y. Coppola J. Yan J. (2022). Single-molecule characterization of subtype-specific beta1 integrin mechanics. Nat. Commun. 13, 7471. 10.1038/s41467-022-35173-w 36463259 PMC9719539

[B60] Jung S. A. Malyaran H. Demco D. E. Manukanc A. Haser L. S. Kucikas V. (2023). Fibrin-dextran hydrogels with tunable porosity and mechanical properties. Biomacromolecules 24, 3972–3984. 10.1021/acs.biomac.3c00269 37574715

[B61] Kantor A. B. Hoffman T. A. M. Mannick D. Lisman T. Huguet J. Offman E. (2025). Safety, tolerability, and pharmacokinetics of single and multiple ascending doses of THN391, a monoclonal antibody targeting the fibrin inflammatory epitope: phase 1a clinical trial results. Alzheimers Dement. 21, e101350. 10.1002/alz70859_101350

[B62] Kaufmanova J. Stikarova J. Hlavackova A. Chrastinova L. Maly M. Suttnar J. (2021). Fibrin clot formation under oxidative stress conditions. Antioxidants (Basel) 10, 923. 10.3390/antiox10060923 34200255 PMC8228070

[B63] Kay A. B. Pepper D. S. McKenzie R. (1974). The identification of fibrinopeptide B as a chemotactic agent derived from human fibrinogen. Br. J. Haematol. 27, 669–677. 10.1111/j.1365-2141.1974.tb06633.x 4472824

[B64] Kearney K. J. Ariëns R. A. S. Macrae F. L. (2022). The role of fibrin(ogen) in wound healing and infection control. Semin. Thromb. Hemost. 48, 174–187. 10.1055/s-0041-1732467 34428799

[B65] Kidwai F. Edwards J. Zou L. Kaufman D. S. (2016). Fibrinogen induces RUNX2 activity and osteogenic development from human pluripotent stem cells. Stem Cells 34, 2079–2089. 10.1002/stem.2427 27331788 PMC5097445

[B66] Kiechl S. Lorenz E. Reindl M. Wiedermann C. J. Oberhollenzer F. Bonora E. (2002). Toll-like receptor 4 polymorphisms and atherogenesis. N. Engl. J. Med. 347, 185–192. 10.1056/NEJMoa012673 12124407

[B67] Kloka J. Friedrichson B. Dauth S. Foldenauer A. C. Bulczak-Schadendorf A. Vehreschild M. (2022). Potential of FX06 to prevent disease progression in hospitalized non-intubated COVID-19 patients - the randomized, EU-wide, placebo-controlled, phase II study design of IXION. Trials 23, 688. 10.1186/s13063-022-06609-x 35986390 PMC9389510

[B68] Kopec A. K. Joshi N. Cline-Fedewa H. Wojcicki A. V. Ray J. L. Sullivan B. P. (2017). Fibrin(ogen) drives repair after acetaminophen-induced liver injury via leukocyte α(M)β(2) integrin-dependent upregulation of Mmp12. J. Hepatol. 66, 787–797. 10.1016/j.jhep.2016.12.004 27965156 PMC5362307

[B69] Kumar L. Planas-Iglesias J. Harms C. Kamboj S. Wright D. Klein-Seetharaman J. (2020). Activity-dependent interdomain dynamics of matrix metalloprotease-1 on fibrin. Sci. Rep. 10, 20615. 10.1038/s41598-020-77699-3 33244162 PMC7692495

[B70] Lawrence S. O. Simpson-Haidaris P. J. (2004). Regulated *de novo* biosynthesis of fibrinogen in extrahepatic epithelial cells in response to inflammation. Thromb. Haemost. 92, 234–243. 10.1160/TH04-01-0024 15269818

[B71] Lee M. E. Rhee K. J. Nham S. U. (1999). Fragment E derived from both fibrin and fibrinogen stimulates interleukin-6 production in rat peritoneal macrophages. Mol. Cells 9, 7–13. 10.1016/S1016-8478(23)13500-X 10102564

[B72] Li R. Liu J. Ma J. Sun X. Wang Y. Yan J. (2022). Fibrinogen improves liver function via promoting cell aggregation and fibronectin assembly in hepatic spheroids. Biomaterials 280, 121266. 10.1016/j.biomaterials.2021.121266 34875515

[B73] Li W. Moretti L. Su X. Yeh C. R. Torres M. P. Barker T. H. (2024). Strain-dependent glutathionylation of fibronectin fibers impacts mechano-chemical behavior and primes an integrin switch. Nat. Commun. 15, 8751. 10.1038/s41467-024-52742-3 39384749 PMC11479631

[B74] Li S. Dan X. Chen H. Li T. Liu B. Ju Y. (2024). Developing fibrin-based biomaterials/scaffolds in tissue engineering. Bioact. Mater 40, 597–623. 10.1016/j.bioactmat.2024.08.006 39239261 PMC11375146

[B75] Lin C. X. Yang K. Li P. C. Gao L. T. Aziz Y. Li J. H. (2024). Self-healing and injectable chitosan/konjac glucomannan hydrogel with pH response for controlled protein release. Colloids Surf. B Biointerfaces 242, 114089. 10.1016/j.colsurfb.2024.114089 39047642

[B76] Liu Y. Li L. Li X. Cherif H. Jiang S. Ghezelbash F. (2024). Viscoelastic hydrogels regulate adipose-derived mesenchymal stem cells for nucleus pulposus regeneration. Acta Biomater. 180, 244–261. 10.1016/j.actbio.2024.04.017 38615812

[B77] Liu Y. Zhang X. Gu W. Su H. Wang X. Wang X. (2025). Unlocking the crucial role of cancer-associated fibroblasts in tumor metastasis: mechanisms and therapeutic prospects. J. Adv. Res. 71, 399–413. 10.1016/j.jare.2024.05.031 38825314 PMC12126706

[B78] Ma S. Wang L. (2024). Fibrinogen-to-albumin ratio (FAR) is the best biomarker for the overall survival of patients with non-small-cell lung cancer. Front. Oncol. 14, 1396843. 10.3389/fonc.2024.1396843 38978733 PMC11228243

[B79] Ma W. Yang S. Wang T. Zhang D. Wu H. Fu S. (2026). Direct extracellular matrix modulation attenuates intestinal fibrosis via a fibronectin-targeted approach. Adv. Sci. (Weinh) 13, e19433. 10.1002/advs.202519433 41616092 PMC13067841

[B80] Maciel J. Oliveira M. I. Colton E. McNally A. K. Oliveira C. Anderson J. M. (2014). Adsorbed fibrinogen enhances production of bone- and angiogenic-related factors by monocytes/macrophages. Tissue Eng. Part A 20, 250–263. 10.1089/ten.TEA.2012.0439 23937279 PMC3875152

[B81] Makowski G. S. Ramsby M. L. (1999). Amorphous calcium phosphate-mediated binding of matrix Metalloproteinase-9 to fibrin is inhibited by pyrophosphate and bisphosphonate. Inflammation 23, 333–360. 10.1023/a:1020209616428 10443797

[B82] Mattiassi S. Conner A. A. Feng F. Goh E. L. K. Yim E. K. F. (2023). The combined effects of topography and stiffness on neuronal differentiation and maturation using a hydrogel platform. Cells 12, 934. 10.3390/cells12060934 36980275 PMC10047827

[B83] McGettigan B. M. Shah V. H. (2024). Every sheriff needs a deputy: targeting non-parenchymal cells to treat hepatic fibrosis. J. Hepatol. 81, 20–22. 10.1016/j.jhep.2024.04.015 38677654

[B84] Medcalf R. L. Keragala C. B. (2021). Fibrinolysis: a primordial system linked to the immune response. Int. J. Mol. Sci. 22, 3406. 10.3390/ijms22073406 33810275 PMC8037105

[B85] Medrano-Bosch M. Simón-Codina B. Jiménez W. Edelman E. R. Melgar-Lesmes P. (2023). Monocyte-endothelial cell interactions in vascular and tissue remodeling. Front. Immunol. 14, 1196033. 10.3389/fimmu.2023.1196033 37483594 PMC10360188

[B86] Minciacchi V. R. Bravo J. Karantanou C. Pereira R. S. Zanetti C. Kumar R. (2024). Exploitation of the fibrinolytic system by B-cell acute lymphoblastic leukemia and its therapeutic targeting. Nat. Commun. 15, 10059. 10.1038/s41467-024-54361-4 39567540 PMC11579293

[B87] Miyamoto S. Nagano Y. Miyazaki M. Nagamura Y. Sasaki K. Kawamura T. (2022). Integrin alpha5 mediates cancer cell-fibroblast adhesion and peritoneal dissemination of diffuse-type gastric carcinoma. Cancer Lett. 526, 335–345. 10.1016/j.canlet.2021.11.008 34775002

[B88] Mo K. Wang Y. Lu C. Li Z. (2024). Insight into the role of macrophages in periodontitis restoration and development. Virulence 15, 2427234. 10.1080/21505594.2024.2427234 39535076 PMC11572313

[B89] Monaco S. Gioia M. Rodriguez J. Fasciglione G. F. Di Pierro D. Lupidi G. (2007). Modulation of the proteolytic activity of matrix metalloproteinase-2 (gelatinase A) on fibrinogen. Biochem. J. 402, 503–513. 10.1042/BJ20061064 17087661 PMC1863560

[B90] Morimatsu M. Mekhdjian A. H. Adhikari A. S. Dunn A. R. (2013). Molecular tension sensors report forces generated by single integrin molecules in living cells. Nano Lett. 13, 3985–3989. 10.1021/nl4005145 23859772 PMC3815579

[B91] Nencini F. Bettiol A. Argento F. R. Borghi S. Giurranna E. Emmi G. (2024). Post-translational modifications of fibrinogen: implications for clotting, fibrin structure and degradation. Mol. Biomed. 5, 45. 10.1186/s43556-024-00214-x 39477884 PMC11525374

[B92] Nencini F. Giurranna E. Borghi S. Taddei N. Fiorillo C. Becatti M. (2025). Fibrinogen oxidation and thrombosis: shaping structure and function. Antioxidants (Basel) 14, 390. 10.3390/antiox14040390 40298646 PMC12024030

[B93] Nguyen M. D. Simpson-Haidaris P. J. (2000). Cell type-specific regulation of fibrinogen expression in lung epithelial cells by dexamethasone and interleukin-1beta. Am. J. Respir. Cell Mol. Biol. 22, 209–217. 10.1165/ajrcmb.22.2.3746 10657942

[B94] Patten J. Halligan P. Bashiri G. Kegel M. Bonadio J. D. Wang K. (2025). EDA fibronectin microarchitecture and YAP translocation during wound closure. ACS Biomater. Sci. Eng. 11, 2249–2262. 10.1021/acsbiomaterials.4c02019 40029610 PMC12426948

[B95] Pechlivani N. Kearney K. J. Ajjan R. A. (2021). Fibrinogen and antifibrinolytic proteins: interactions and future therapeutics. Int. J. Mol. Sci. 22, 12537. 10.3390/ijms222212537 34830419 PMC8625824

[B96] Peng Z. Hao M. Tong H. Yang H. Huang B. Zhang Z. (2022). The interactions between integrin alpha(5)beta(1) of liver cancer cells and fibronectin of fibroblasts promote tumor growth and angiogenesis. Int. J. Biol. Sci. 18, 5019–5037. 10.7150/ijbs.72367 35982891 PMC9379399

[B97] Pereira M. Rybarczyk B. J. Odrljin T. M. Hocking D. C. Sottile J. Simpson-Haidaris P. J. (2002). The incorporation of fibrinogen into extracellular matrix is dependent on active assembly of a fibronectin matrix. J. Cell Sci. 115, 609–617. 10.1242/jcs.115.3.609 11861767

[B98] Pereira R. V. S. EzEldeen M. Ugarte-Berzal E. Martens E. Malengier-Devlies B. Vandooren J. (2023). Physiological fibrin hydrogel modulates immune cells and molecules and accelerates mouse skin wound healing. Front. Immunol. 14, 1170153. 10.3389/fimmu.2023.1170153 37168862 PMC10165074

[B99] Perutelli P. Marchese P. Mori P. G. (1992). The glycoprotein IIb/IIIa complex of the platelets. An activation-dependent integrin. Recenti Prog. Med. 83, 100–104. 1323869

[B100] Petzelbauer P. Zacharowski P. A. Miyazaki Y. Friedl P. Wickenhauser G. Castellino F. J. (2005). The fibrin-derived peptide Bbeta15-42 protects the myocardium against ischemia-reperfusion injury. Nat. Med. 11, 298–304. 10.1038/nm1198 15723073

[B101] Piglionico S. S. Varga B. Pall O. Romieu O. Gergely C. Cuisinier F. (2023). Biomechanical characterization of a fibrinogen-blood hydrogel for human dental pulp regeneration. Biomater. Sci. 11, 6919–6930. 10.1039/d3bm00515a 37655620

[B102] Polzin A. Benkhoff M. Thienel M. Barcik M. Mourikis P. Shchurovska K. (2025). Long-term FXa inhibition attenuates thromboinflammation after acute myocardial infarction and stroke by platelet proteome alteration. J. Thromb. Haemost. 23, 668–683. 10.1016/j.jtha.2024.10.025 39551435

[B103] Poole L. G. Pant A. Baker K. S. Kopec A. K. Cline‐Fedewa H. M. Iismaa S. E. (2019). Chronic liver injury drives non‐traditional intrahepatic fibrin(ogen) crosslinking via tissue transglutaminase. J. Thrombosis Haemostasis 17, 113–125. 10.1111/jth.14330 30415489 PMC6322974

[B104] Poole L. G. Kopec A. K. Groeneveld D. J. Pant A. Baker K. S. Cline-Fedewa H. M. (2021). Factor XIII cross-links fibrin(ogen) independent of fibrin polymerization in experimental acute liver injury. Blood 137, 2520–2531. 10.1182/blood.2020007415 33569603 PMC8109015

[B105] Ramos-DeSimone N. Hahn-Dantona E. Sipley J. Nagase H. French D. L. Quigley J. P. (1999). Activation of matrix Metalloproteinase-9 (MMP-9) via a converging Plasmin/Stromelysin-1 Cascade enhances tumor cell invasion. J. Biol. Chem. 274, 13066–13076. 10.1074/jbc.274.19.13066 10224058

[B106] Rao S. V. Kirsch B. Bhatt D. L. Budaj A. Coppolecchia R. Eikelboom J. (2022). A multicenter, phase 2, randomized, placebo-controlled, double-blind, parallel-group, dose-finding trial of the oral factor XIa inhibitor Asundexian to prevent adverse cardiovascular outcomes after acute myocardial infarction. Circulation 146, 1196–1206. 10.1161/circulationaha.122.061612 36030390

[B107] Reyhani V. Seddigh P. Guss B. Gustafsson R. Rask L. Rubin K. (2014). Fibrin binds to collagen and provides a bridge for αVβ3 integrin-dependent contraction of collagen gels. Biochem. J. 462, 113–123. 10.1042/BJ20140201 24840544 PMC4109839

[B108] Riedel T. Suttnar J. Brynda E. Houska M. Medved L. Dyr J. E. (2011). Fibrinopeptides A and B release in the process of surface fibrin formation. Blood 117, 1700–1706. 10.1182/blood-2010-08-300301 21106983 PMC3056594

[B109] Risman R. A. Sen M. Tutwiler V. Hudson N. E. (2025). Deconstructing fibrin(ogen) structure. J. Thromb. Haemost. 23, 368–380. 10.1016/j.jtha.2024.10.024 39536819 PMC11786978

[B110] Rudnik M. Hukara A. Kocherova I. Jordan S. Schniering J. Milleret V. (2021). Elevated fibronectin levels in profibrotic CD14(+) monocytes and CD14(+) macrophages in systemic sclerosis. Front. Immunol. 12, 642891. 10.3389/fimmu.2021.642891 34504485 PMC8421541

[B111] Rybarczyk B. J. Simpson-Haidaris P. J. (2000). Fibrinogen assembly, secretion, and deposition into extracellular matrix by MCF-7 human breast carcinoma cells. Cancer Res. 60, 2033–2039. 10766195

[B112] Ryu J. K. Rafalski V. A. Meyer-Franke A. Adams R. A. Poda S. B. Rios Coronado P. E. (2018). Fibrin-targeting immunotherapy protects against neuroinflammation and neurodegeneration. Nat. Immunol. 19, 1212–1223. 10.1038/s41590-018-0232-x 30323343 PMC6317891

[B113] Sahni A. Simpson-Haidaris P. J. Sahni S. K. Vaday G. G. Francis C. W. (2008). Fibrinogen synthesized by cancer cells augments the proliferative effect of fibroblast growth factor-2 (FGF-2). J. Thromb. Haemost. 6, 176–183. 10.1111/j.1538-7836.2007.02808.x 17949478

[B114] Santos S. G. Lamghari M. Almeida C. R. Oliveira M. I. Neves N. Ribeiro A. C. (2013). Adsorbed fibrinogen leads to improved bone regeneration and correlates with differences in the systemic immune response. Acta Biomater. 9, 7209–7217. 10.1016/j.actbio.2013.04.008 23571000

[B115] Sanz-Horta R. Matesanz A. Gallardo A. Reinecke H. Jorcano J. L. Acedo P. (2023). Technological advances in fibrin for tissue engineering. J. Tissue Eng. 14, 20417314231190288. 10.1177/20417314231190288 37588339 PMC10426312

[B116] Sarkar A. Niraula G. LeVine D. Zhao Y. Tu Y. Mollaeian K. (2023). Development of a ratiometric tension sensor exclusively responding to integrin tension magnitude in live cells. ACS Sens. 8, 3701–3712. 10.1021/acssensors.3c00606 37738233 PMC10788086

[B117] Seltana A. Cloutier G. Reyes Nicolas V. Khalfaoui T. Teller I. C. Perreault N. (2022). Fibrin(ogen) is constitutively expressed by differentiated intestinal epithelial cells and mediates wound healing. Front. Immunol. 13, 916187. 10.3389/fimmu.2022.916187 35812445 PMC9258339

[B118] Shabayek S. Spellerberg B. (2018). Group B streptococcal colonization, molecular characteristics, and epidemiology. Front. Microbiol. 9, 437. 10.3389/fmicb.2018.00437 29593684 PMC5861770

[B119] Shepherd J. Bax D. Best S. Cameron R. (2017). Collagen-fibrinogen lyophilised scaffolds for soft tissue regeneration. Materials (Basel) 10 (6), 568. 10.3390/ma10060568 28772927 PMC5541296

[B120] Simpson-Haidaris P. J. Rybarczyk B. (2001). Tumors and fibrinogen. The role of fibrinogen as an extracellular matrix protein. Ann. N. Y. Acad. Sci. 936, 406–425. 10.1111/j.1749-6632.2001.tb03525.x 11460495

[B121] Sims G. P. Scaletta L. Andrews J. Sims D. A. Strain M. Sigurdardottir A. (2026). Preclinical characterization of AZD1163, a first-in-class anti-PAD2/4 bispecific antibody for the treatment of rheumatoid arthritis. MAbs 18, 2657629. 10.1080/19420862.2026.2657629 41982149 PMC13085943

[B122] Sinha S. Ayushman M. Tong X. Yang F. (2023). Dynamically crosslinked poly(ethylene-glycol) hydrogels reveal a critical role of viscoelasticity in modulating glioblastoma fates and drug responses in 3D. Adv. Healthc. Mater 12, e2202147. 10.1002/adhm.202202147 36239185 PMC9813196

[B123] Staton C. A. Brown N. J. Rodgers G. R. Corke K. P. Tazzyman S. Underwood J. C. (2004). Alphastatin, a 24-amino acid fragment of human fibrinogen, is a potent new inhibitor of activated endothelial cells *in vitro* and *in vivo* . Blood 103, 601–606. 10.1182/blood-2003-07-2192 14512300

[B124] Sulimai N. Brown J. Lominadze D. (2021). The effects of fibrinogen's interactions with its neuronal receptors, intercellular adhesion Molecule-1 and cellular prion protein. Biomolecules 11, 1381. 10.3390/biom11091381 34572594 PMC8464854

[B125] Sun L. Li J. Gao W. Shi M. Tang F. Fu X. (2021). Coaxial nanofibrous scaffolds mimicking the extracellular matrix transition in the wound healing process promoting skin regeneration through enhancing immunomodulation. J. Mater Chem. B 9, 1395–1405. 10.1039/d0tb01933j 33462572

[B126] Suter N. Joshi A. Wunsch T. Graupner N. Stapelfeldt K. Radmacher M. (2021). Self-assembled fibrinogen nanofibers support fibroblast adhesion and prevent *E. coli* infiltration. Mater Sci. Eng. C Mater Biol. Appl. 126, 112156. 10.1016/j.msec.2021.112156 34082961

[B127] Tenopoulou M. (2025). Fibrinogen post-translational modifications are biochemical determinants of fibrin clot properties and interactions. Febs J. 292, 11–27. 10.1111/febs.17236 39180244 PMC11705221

[B128] Tilvawala R. Nemmara V. V. Reyes A. C. Sorvillo N. Salinger A. J. Cherpokova D. (2021). The role of SERPIN citrullination in thrombosis. Cell Chem. Biol. 28, 1728–1739.e5. 10.1016/j.chembiol.2021.07.009 34352225 PMC8688209

[B129] Ting M. S. Travas-Sejdic J. Malmström J. (2021). Modulation of hydrogel stiffness by external stimuli: soft materials for mechanotransduction studies. J. Mater Chem. B 9, 7578–7596. 10.1039/d1tb01415c 34596202

[B130] Ugarova T. P. Yakubenko V. P. (2001). Recognition of fibrinogen by leukocyte integrins. Ann. N. Y. Acad. Sci. 936, 368–385. 10.1111/j.1749-6632.2001.tb03523.x 11460493

[B131] Vasconcelos D. M. Gonçalves R. M. Almeida C. R. Pereira I. O. Oliveira M. I. Neves N. (2016). Fibrinogen scaffolds with immunomodulatory properties promote *in vivo* bone regeneration. Biomaterials 111, 163–178. 10.1016/j.biomaterials.2016.10.004 27728815

[B132] Wang P. Li L. Zhang C. Lei Q. Fang W. (2010). Effects of fractal surface on C6 glioma cell morphogenesis and differentiation *in vitro* . Biomaterials 31, 6201–6206. 10.1016/j.biomaterials.2010.04.034 20510443

[B133] Wang J. Pathak R. Garg S. Hauer-Jensen M. (2017). Fibrinogen deficiency suppresses the development of early and delayed radiation enteropathy. World J. Gastroenterol. 23, 4701–4711. 10.3748/wjg.v23.i26.4701 28765691 PMC5514635

[B134] Wang K. Mosser G. Haye B. Baccile N. Le Griel P. Pernot P. (2021). Cellulose nanocrystal-fibrin nanocomposite hydrogels promoting myotube formation. Biomacromolecules 22, 2740–2753. 10.1021/acs.biomac.1c00422 34027656

[B135] Wang Y. Wu Y. Long L. Yang L. Fu D. Hu C. (2021). Inflammation-responsive drug-loaded hydrogels with sequential hemostasis, antibacterial, and anti-inflammatory behavior for chronically infected diabetic wound treatment. ACS Appl. Mater Interfaces 13, 33584–33599. 10.1021/acsami.1c09889 34240605

[B136] Wang S. Wang J. Liu C. Yang L. Tan X. Chen S. (2024). Neoplastic ICAM-1 protects lung carcinoma from apoptosis through ligation of fibrinogen. Cell Death Dis. 15, 605. 10.1038/s41419-024-06989-9 39168965 PMC11339363

[B137] Weisel J. W. Litvinov R. I. (2013). Mechanisms of fibrin polymerization and clinical implications. Blood 121, 1712–1719. 10.1182/blood-2012-09-306639 23305734 PMC3591795

[B138] Winkler J. Abisoye-Ogunniyan A. Metcalf K. J. Werb Z. (2020). Concepts of extracellular matrix remodelling in tumour progression and metastasis. Nat. Commun. 11, 5120. 10.1038/s41467-020-18794-x 33037194 PMC7547708

[B139] Wolberg A. S. (2023). Fibrinogen and fibrin: synthesis, structure, and function in health and disease. J. Thromb. Haemost. 21, 3005–3015. 10.1016/j.jtha.2023.08.014 37625698 PMC10592048

[B140] Wu X. Yu X. Chen C. Chen C. Wang Y. Su D. (2024). Fibrinogen and tumors. Front. Oncol. 14, 1393599. 10.3389/fonc.2024.1393599 38779081 PMC11109443

[B141] Xie X. Chen X. Zhou J. Wang T. Yang G. Han F. (2025). Dynamic hydrogels with tunable mechanics for 3D organoid derivation. Small 21, e2501862. 10.1002/smll.202501862 40434214

[B142] Xu Z. Zhang L. Bentil S. A. Bratlie K. M. (2021). Gellan gum-gelatin viscoelastic hydrogels as scaffolds to promote fibroblast differentiation. Mater Sci. Eng. C Mater Biol. Appl. 129, 112370. 10.1016/j.msec.2021.112370 34579889

[B143] Xu M. Du R. Xing W. Chen X. Wan J. Wang S. (2023). Platelets derived citrullinated proteins and microparticles are potential autoantibodies ACPA targets in RA patients. Front. Immunol. 14, 1084283. 10.3389/fimmu.2023.1084283 36761728 PMC9902922

[B144] Yakovlev S. Mikhailenko I. Tsurupa G. Belkin A. M. Medved L. (2014). Polymerisation of fibrin αC-domains promotes endothelial cell migration and proliferation. Thromb. Haemost. 112, 1244–1251. 10.1160/TH14-01-0079 25220673 PMC4406416

[B145] Yakovlev S. Strickland D. K. Medved L. (2022). Current view on the molecular mechanisms underlying fibrin(ogen)-dependent inflammation. Thromb. Haemost. 122, 1858–1868. 10.1055/a-1910-4538 35896433 PMC10680782

[B146] Yuan T. T. DiGeorge Foushee A. M. Johnson M. C. Jockheck-Clark A. R. Stahl J. M. (2018). Development of electrospun chitosan-polyethylene oxide/fibrinogen biocomposite for potential wound healing applications. Nanoscale Res. Lett. 13, 88. 10.1186/s11671-018-2491-8 29611009 PMC5880797

[B147] Zanut A. Li R. Deng R. Liu X. Rejhon M. Chen W. (2023). A polymer canvas with the stiffness of the bone matrix to study and control mesenchymal stem cell response. Adv. Healthc. Mater 12, e2201503. 10.1002/adhm.202201503 36565136

[B148] Zeng Z. Li M. Zhu S. Zhang K. Wu Y. Zheng M. (2024). Strain-level genomic analysis of serotype, genotype and virulence gene composition of group B streptococcus. Front. Cell Infect. Microbiol. 14, 1396762. 10.3389/fcimb.2024.1396762 39569407 PMC11576427

[B149] Zhang X. Long Q. (2017). Elevated serum plasma fibrinogen is associated with advanced tumor stage and poor survival in hepatocellular carcinoma patients. Med. Baltim. 96, e6694. 10.1097/MD.0000000000006694 28445272 PMC5413237

[B150] Zhang C. Chen H. He Q. Luo Y. He A. Tao A. (2021). Fibrinogen/AKT/Microfilament axis promotes colitis by enhancing vascular permeability. Cell Mol. Gastroenterol. Hepatol. 11, 683–696. 10.1016/j.jcmgh.2020.10.007 33075564 PMC7843406

[B151] Zhang Z. Zhu H. Zhao G. Miao Y. Zhao L. Feng J. (2023). Programmable and reversible integrin-mediated cell adhesion reveals hysteresis in actin kinetics that alters subsequent mechanotransduction. Adv. Sci. (Weinh) 10, e2302421. 10.1002/advs.202302421 37849221 PMC10724447

[B152] Zhang M. Yu T. Li J. Yan H. Lyu L. Yu Y. (2024). Matrix metalloproteinase-responsive hydrogel with On-Demand release of phosphatidylserine promotes bone regeneration through immunomodulation. Adv. Sci. (Weinh) 11, e2306924. 10.1002/advs.202306924 38460178 PMC11132073

[B153] Zhang Y. Li Z. Zhang J. Mafa T. Zhang J. Zhu H. (2025). Fibrinogen: a new player and target on the formation of pre-metastatic niche in tumor metastasis. Crit. Rev. Oncol. Hematol. 207, 104625. 10.1016/j.critrevonc.2025.104625 39826884

[B154] Zhao P. Sun T. Lyu C. Liang K. Du Y. (2023). Cell mediated ECM-Degradation as an emerging tool for anti-fibrotic strategy. Cell Regen. 12, 29. 10.1186/s13619-023-00172-9 37653282 PMC10471565

[B155] Zhao S. Xue C. Burns D. C. Shoichet M. S. (2024). Viscoelastic supramolecular hyaluronan-peptide cross-linked hydrogels. Biomacromolecules 25, 3946–3958. 10.1021/acs.biomac.4c00095 38913947

[B156] Zheng D. W. Hong S. Zhang Q. L. Dong X. Pan P. Song W. F. (2020). Controllable gelation of artificial extracellular matrix for altering mass transport and improving cancer therapies. Nat. Commun. 11, 4907. 10.1038/s41467-020-18493-7 32999289 PMC7527557

[B157] Zheng Z. Sun L. Li Y. Wang S. Wang P. Qiu S. (2025). Gelatin-dopamine-based dual-responsive nanogels for tumor-targeted bortezomib delivery: minimizing systemic toxicity and enhancing breast cancer therapy. Int. J. Biol. Macromol. 318, 145084. 10.1016/j.ijbiomac.2025.145084 40494466

[B158] Zuo H. Jiao Y. Chen J. Tong S. Li Y. Zhao W. (2026). Recent advances in smart stimulus-responsive hydrogels for precision drug delivery in tumours. Gels 12, 98. 10.3390/gels12020098 41744970 PMC12941131

